# QTL rich genomic clusters in wheat: a catalogue for resistance to *Puccinia striiformis* f. sp. *tritici*

**DOI:** 10.3389/fpls.2026.1864074

**Published:** 2026-08-04

**Authors:** Cyrus Micheni, Davinder Singh, Robert F. Park, Laura Ziems

**Affiliations:** The Plant Breeding Institute, University of Sydney, Cobbitty, NSW, Australia

**Keywords:** IWGSC RefSeq v2.1, QTL rich clusters, resistance, stripe rust, wheat, wheat genome

## Abstract

Wheat stripe/yellow rust, caused by *Puccinia striiformis* f. sp. *tritici* (Pst), remains one of the most significant constraints to global wheat production. Genetic resistance breeding is the most effective, economical, and environmentally sustainable strategy for controlling stripe rust. Over the past two decades, advances in genome sequencing, molecular markers and high-throughput genotyping have led to the reporting of numerous *Yr* genes and an even greater number of quantitative trait loci (QTL, *QYr*). Alignment of *QYr* onto a common refence map is therefore required to consolidate dispersed reports, identify regions of co-localization, and define stable genomic regions underlying stripe rust resistance. In this review, catalogued *Yr* genes and published stripe rust QTL were systematically compiled from 142 studies and, where sufficient marker information was available, physically re-anchored to the IWGSC RefSeq v2.1 wheat reference genome. This integrative approach enabled consolidation of overlapping loci and delineation of 42 QTL-rich clusters (QRCs). The B-sub genome had the highest number of clusters (20), followed by the A-sub genome (16). The D-sub genome had the least number of clusters (6), with two chromosomes, 5D and 6D, lacking any clusters. Thirteen cloned *Yr* genes were mapped within these clusters, thereby providing functional validation of QRCs as biologically meaningful reservoirs of resistance. This cluster-based framework provides a practical roadmap for consolidating *Yr/QYr* loci, prioritizing novel genomic targets, and deploying resistance strategically against evolving pathogen populations.

## 1 Introduction

Wheat production faces numerous constraints that threaten its sustainability and productivity, including both biotic and abiotic stresses (Mao et al., 2023). Among the biotic stresses, fungal diseases, particularly rusts, are a major concern (Bhardwaj et al., 2019). Wheat is vulnerable to three major rust diseases: stem rust (caused by *Puccinia graminis* f. sp. *tritici*; Pgt), leaf rust (*Puccinia triticina*; Pt), and stripe rust (*Puccinia striiformis* f. sp. *tritici*; Pst) (Singh et al., 2024a). These fungal pathogens cause the most destructive diseases of wheat, resulting in significant yield losses and economic damage worldwide (Singh et al., 2024a).

Of the three rust diseases of wheat, stripe rust is the most prevalent and destructive worldwide. It is characterized by bright yellow-orange pustules arranged in linear stripes along the leaves, although it can also infect stems, spikes, and grains under severe conditions (Wellings, 2007). The economic impact of stripe rust is substantial, with yield losses ranging from 5% to 90% in susceptible varieties, depending on disease severity and environmental conditions (Chen et al., 2014). Epidemics can lead to significant reductions in grain quality and quantity, resulting in economic losses for farmers and increased food prices for consumers (Line, 2002). In recent decades, the emergence of aggressive Pst races and changing climatic conditions have exacerbated the threat of stripe rust, particularly in regions where it was previously less prevalent (Zhang et al., 2024). While chemical control provides short-term relief, genetic resistance remains the cornerstone of sustainable rust management, offering long-term protection and reducing reliance on fungicides that have economic and environmental impacts (Paul et al., 2025).

The genetic basis of host resistance to rust diseases comprises both major (qualitative) and minor (quantitative) resistance genes (Ziems et al., 2017). Major resistance genes, commonly referred to as all-stage resistance (ASR), confer rapid and often complete protection against specific rust pathotypes and are expressed from the seedling stage through plant maturity (Bariana, 2003). This form of resistance is classically explained by Flor’s gene-for-gene model, in which a host resistance (R) gene recognizes a corresponding avirulence (Avr) effector from the pathogen, triggering a hypersensitive response characterized by localized cell death that restricts pathogen growth (Flor, 1955; McIntosh et al., 1995; Choi et al., 2021). Given the ease of detecting ASR in standard seedling assays, these genes formed the foundation of early rust resistance breeding programs (Johnson, 1992). However, the tendency of ASR to be race-specific imposes intense selection pressure on pathogen populations, often resulting in the rapid emergence of virulent races and the well-documented boom-and-bust cycle (Park, 2008; Hovmøller et al., 2015; Singh et al., 2024a). Consequently, many ASR genes show limited effectiveness across diverse geographical regions, and if they are relied on as the sole source of resistance, the developed germplasm is vulnerable. The widely deployed *Yr15* illustrates this risk, with virulence recently detected in Europe and key cultivars now rendered susceptible (Davis et al., 2025).

In contrast, minor resistance genes typically confer partial, race-nonspecific resistance and are quantitative in nature, often involving the cumulative effects of multiple loci (Singh et al., 2004; Ziems et al., 2017). These genes underpin adult plant resistance (APR), which is typically expressed only after the seedling stage; generally from tillering or stem elongation onward, influenced by environmental and physiological factors, and persists throughout the crop cycle (Park and Rees, 1989). APR is phenotypically characterized by reduced disease severity, smaller pustules, prolonged latent period and increased chlorosis or necrosis, resulting in a “slow-rusting” response that delays epidemic development rather than preventing infection (Mares and Cousen, 1977; Ma and Singh, 1996; Herrera-Foessel et al., 2006). Because APR does not exert strong selection pressure on pathogen populations, it is considered durable and forms the basis of long-term resistance strategies (Ellis et al., 2014; Mundt, 2018; Dinglasan et al., 2022). Detection of APR typically requires phenotyping at the adult growth stage under field conditions, as expression is often development-regulated and environment-dependent (Rothwell et al., 2018; Gill et al., 2025). Consequently, reliable screening under controlled conditions can be challenging because the specific environmental cues required to activate certain APR loci are almost impossible to reproduce (Rothwell et al., 2018).

Within the APR spectrum, additional specialized resistance forms have been identified, including high-temperature adult plant resistance (HTAPR) and race-specific APR. Stripe rust has been regarded historically as a disease adapted to cool, moist environments, with optimal development between 10–16 °C and suppression at temperatures above 20 °C (Park, 1990). However, Pst has evolved increased thermal tolerance, enabling infection, sporulation, and survival at temperatures exceeding 18 °C and during short heat stress episodes up to 30–35 °C (Milus et al., 2009; Chen, 2013). HTAPR exploits this thermal dependency by conferring resistance only under elevated daytime temperatures, typically above 25 °C, while remaining inactive under cooler conditions (<20 °C), rendering seedlings susceptible in standard assays (Milus et al., 2009; Chen, 2013). HTAPR is considered race-nonspecific, significantly reducing disease severity at the adult plant stage (Carter et al., 2009; Chen, 2013). Race-specific APR is also expressed only after the seedling stage and presents as a partial slow-rusting phenotype or, in some cases, a relatively strong adult-stage resistance response but it is ultimately ineffective against virulent pathotypes, leading to complete resistance breakdown (Ellis et al., 2014; Milus et al., 2015; Singh et al., 2015). Race-specific APR is distinguished by susceptibility in seedling assays, adult-stage expression of resistance, and loss of effectiveness when challenged by virulent isolates (Milus et al., 2009; Riaz et al., 2016).

Achieving durable resistance is a key goal in rust management, as it provides long-term protection against a broad range of pathogen races (Ziems et al., 2014). One strategy to achieve durable resistance is gene pyramiding, which involves stacking multiple resistance genes (both ASR and APR) in a single cultivar (Mundt, 2018). This approach makes it more difficult for the pathogen to overcome resistance, as it would need to evade multiple defense mechanisms simultaneously (Lof et al., 2017). Gene pyramiding has been successfully used in breeding programs to develop wheat cultivars with enhanced and durable resistance to rust diseases (Aravindh et al., 2020).

The development of molecular marker technologies has fundamentally transformed host genetics research and wheat breeding by enabling the precise identification, mapping, and deployment of genes associated with economically important traits, including resistance to Pst (Singh et al., 2024a, 2024b; Tong et al., 2024). Simple Sequence Repeats (SSRs) to high-density Single Nucleotide Polymorphism (SNP) arrays and sequencing-based platforms, have significantly enhanced the detection and mapping of stripe rust resistance loci in wheat (Mir et al., 2013; Rimbert et al., 2018). These innovations have facilitated high-resolution linkage mapping, Genome-Wide Association Studies (GWAS), and marker-assisted breeding, thereby substantially increasing the number of reported *Yr* genes and Quantitative Trait Loci (QTLs) (Bokore et al., 2017; Cui et al., 2017; Pandey et al., 2017; Pradhan et al., 2020; Huang et al., 2021; Abdi et al., 2026). The integration of these marker platforms with high-quality wheat reference genomes, notably the IWGSC RefSeq assemblies (v1.0 and v2.1; International Wheat Genome Sequencing, 2018), has improved the resolution and accuracy of stripe rust resistance mapping (International Wheat Genome Sequencing, 2018; Zhu et al., 2021; Tong et al., 2024). Anchoring markers to physical genome coordinates has enabled the development of high-density reference genomes and facilitated genome-wide association studies (GWAS), allowing precise localization of resistance genes and quantitative trait loci (QTL) across diverse germplasm (Jannink et al., 2010).

To date, 87 stripe rust resistance (*Yr*) genes have been catalogued, comprising approximately 62 ASR genes and 25 APR genes, the latter of which include HTAPR and pleiotropic loci. These genes exhibit variable effectiveness against contemporary Pst pathotypes, with several displaying pleiotropic effects or tight linkage that confer resistance to multiple rust species (McIntosh, 2024; Qureshi et al., 2025). Beyond these formally designated genes, extensive quantitative variation for stripe rust resistance has been documented across global wheat germplasm. High-density mapping has led to the identification of more than 500 *Yr* QTL over the past decade (Kumar et al., 2023; Tong et al., 2024), representing an approximately five-fold increase compared with the 140 QTL summarized in earlier reviews (Rosewarne et al., 2013). Many of these QTL likely represent identical genes spread through breeding programs. This repeat identification stems from several key factors, including population specific genetic maps, the use of different marker systems, and varying reference genomes, which pose a challenge for recognizing whether a locus has already been reported. In addition, many mapping populations share common parental lines for several reasons, including the availability of reference maps or as standard crossing backgrounds contributing to the recurrent detection of identical loci under different QTL names (Jansen et al., 2003; Rosewarne et al., 2013). Regions of low recombination and large introgressed segments, further constrain mapping resolution and inflates confidence intervals, increasing the likelihood that closely linked loci are reported as distinct QTL (Cockram and Mackay, 2018).

This review aims to physically re-anchor stripe rust resistance loci, including catalogued *Yr* genes and QTL, onto the IWGSC RefSeq v2.1 Chinese Spring assembly to resolve genomic clusters of stripe rust resistance and identify underutilized regions. QTL-rich cluster (QRC) were defined by a genomic region containing three or more physically anchored stripe rust resistance QTL within a rolling 20 Mb framework. Co-localized loci were defined by *Yr* genes and/or *QYr* positioned within the same or overlapping physical region, without implying allelism or a shared causal gene. By consolidating studies into a unified physical framework, this synthesis makes it easier to compare results across studies, recognize when newly identified loci overlap with previously reported resistance regions, and more confidently assess locus novelty. It also highlights genomic regions that have been repeatedly associated with stripe rust resistance but remain poorly characterized, helping to prioritize future research targets and providing a breeder-ready roadmap to guide the strategic deployment and pyramiding of durable stripe rust resistance in wheat.

## 2 QTL-rich genomic regions underlying stripe rust resistance in wheat

### 2.1 Compilation and physical anchoring of *Yr*/*QYr* loci

To compile comprehensive and accurate information on catalogued *Yr* genes and reported *QYr* loci, data were collated from the Catalogue of Gene Symbols for Wheat (2024 edition) and an extensive body of peer-reviewed literature. The Catalogue provided standardized information on *Yr* loci, including gene nomenclature, donor sources, and chromosomal assignments. In addition, data on *Yr*/*QYr* were extracted from 142 historical *Yr* resistance mapping studies published between 2000 and 2023 (**Supplementary Table 1**). These studies identified predominantly resistance loci under field conditions using natural or artificial Pst infection. Given the extensive volume of published literature on stripe rust resistance, inclusion was restricted to studies reporting sufficient genetic, phenotypic, and mapping information to enable reliable locus comparison and physical positioning; studies lacking relevant or interpretable data were therefore excluded from the synthesis.

Sequence information associated with reported markers was collected from the publications or sourced from GrainGenes (Yao et al., 2022), CerealsDB (Wilkinson et al., 2012) or the DArT website (https://www.diversityarrays.com/resources/download-genetic-maps/). Information was sourced from Roder et al. (1998) and Cui et al. (2017) for SSR and Wheat660K SNP array sequences, respectively. BLAST functions in GrainGenes (Yao et al., 2022) and WheatOmics 1.0 (Ma et al., 2021) were used to identify the mid-point location based on the IWGSC RefSeq v2.1 reference genome (Zhu et al., 2021). Data was collated systematically, and cross-referencing was performed to ensure accuracy and consistency, with discrepancies resolved by comparing multiple literature sources. Where physical positions could not be independently validated through marker anchoring, published IWGSC RefSeq v2.1 positions were retained from the original study or associated references, including Tong et al. (2024). **Supplementary Table 1** provides a comprehensive catalogue of *Yr*/*QYr*, including their chromosomal assignments, closely linked or flanking molecular markers, and corresponding physical mid-point positions anchored to the wheat reference genome. This table consolidates dispersed literature into a unified genomic framework, facilitating direct comparison among loci and supporting the identification of resistance clusters. Cluster IDs in **Supplementary Table 1** indicate loci assigned to QRCs using the rolling 20 Mb threshold; loci without a cluster assignment were retained in the table for completeness but were not considered part of a QRC.

### 2.2 Definition of QTL-rich clusters

To identify *QYr* rich regions, QTL were ordered by physical position within each chromosome and grouped using a 20 Mb sliding window (or rolling threshold) approach. QTL were considered part of the same cluster when successive loci from different studies were located within 20 Mb of one another. Once a QTL was added, the 20 Mb search interval was extended from that position, and clustering continued until no further QTL were captured. Where adjacent 20 Mb windows overlapped, QRC were merged. A new QRC was initiated only when the distance between two successive projected loci exceeded 20 Mb. Previous studies have classified QTL-rich clusters (QRCs) using a narrower threshold of less than 10 Mb between QTL identified in historical mapping studies (Xu et al., 2023; Tong et al., 2024). The broader confidence limit (20 Mb) used here was intended to account for differences arising from projection across different reference genomes and errors in QTL mapping studies while also reducing fragmentation of nearby loci into multiple clusters within the same region. The 20 Mb threshold was evaluated using a sensitivity check with narrower sliding window of 10 and 15 Mb. Although the 10 Mb threshold produced the same total number of QRCs as the 20 Mb threshold, these were distributed across fewer chromosomes (15/21 compared with 19/21), showed greater local concentration on individual chromosomes (maximum of seven QRCs on one chromosome compared with five at 20 Mb), and retained fewer loci within QRCs (241 compared with 309). Similarly, the 15 Mb threshold produced fewer QRCs overall (40), distributed across 17 chromosomes, and retained 272 loci. In contrast, the 20 Mb threshold identified 42 QRCs distributed across 19 of the 21 wheat chromosomes, with a median of two QRCs per chromosome and a maximum of five QRCs on any individual chromosome. This indicated that the 20 Mb threshold retained a broader and more biologically interpretable genome-wide distribution while avoiding excessive fragmentation of recurrent resistance regions.

### 2.3 Genome-wide distribution of QRCs

**Figure 1:**
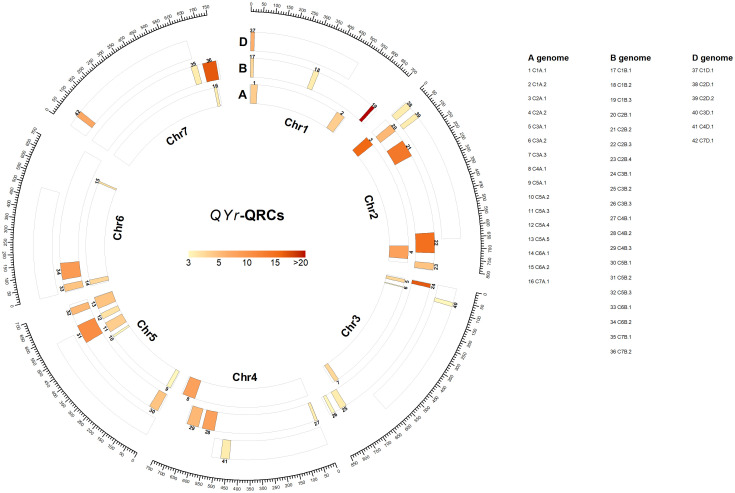
Circular heatmap showing the number of QTL contributing to each QRC and their physical distribution along wheat homologous chromosomes (A, B and D sub-genomes). Regions with greater colour intensity indicate areas with a higher concentration of previously reported QTL. Physical positions of all QTL regions are provided in **Supplementary file 1**, **Table S2**.

**Figure 2:**
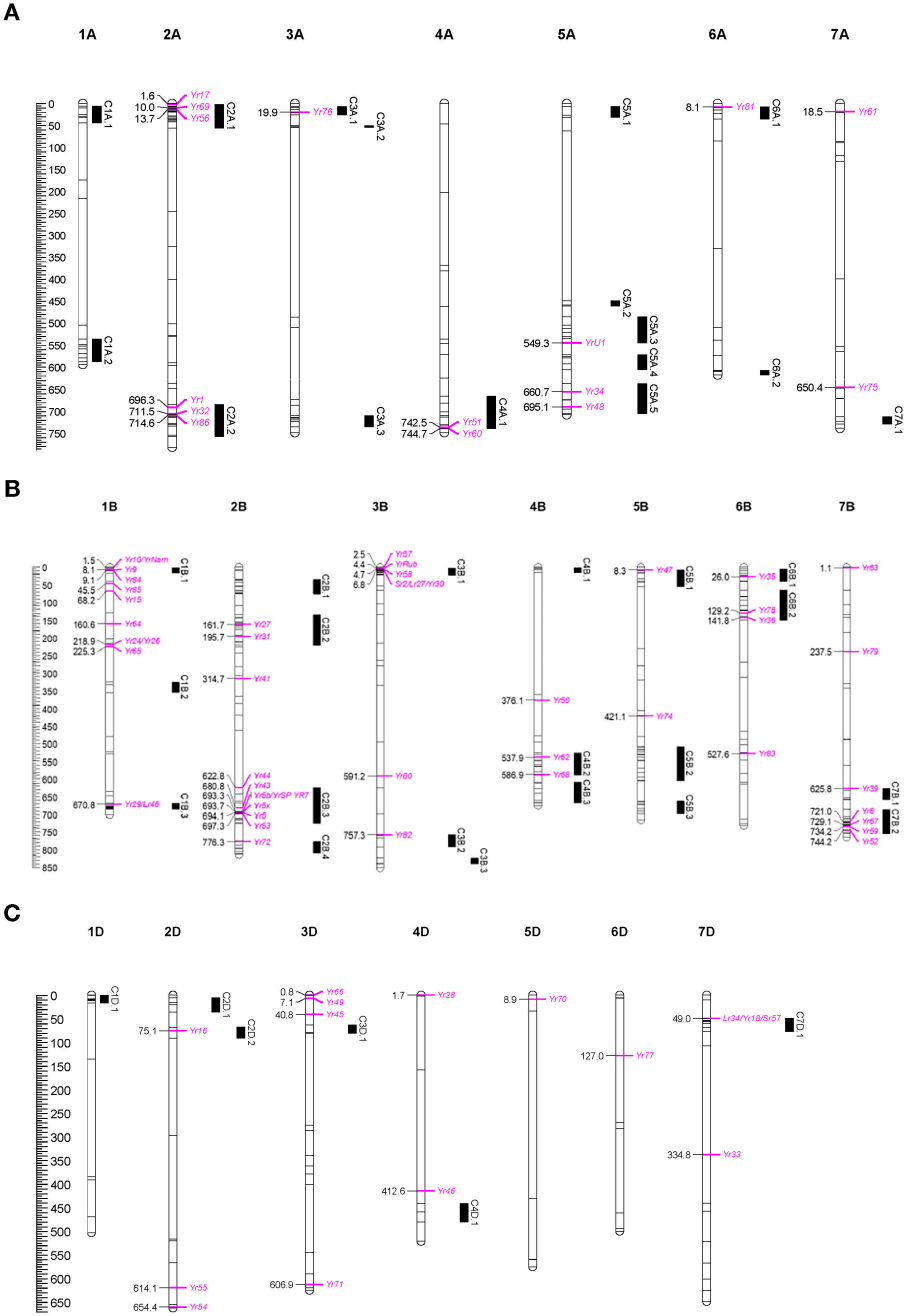
QTL-rich clusters (QRCs) identified on the wheat **(A)** A, **(B)** B, and **(C)** D sub-genomes based on physically anchored *QYr* positions on the IWGSC RefSeq v2.1 assembly. Known *Yr* genes are shown in pink to highlight co-location between QRCs and characterised stripe rust resistance loci. Horizontal lines on the chromosomes indicate individual reported *QYr* positions, including loci not assigned to a QRC. Physical positions of all QTL regions are provided in **Supplementary file 1**, **Table S2**.

Of the 87 catalogued *Yr* genes, 64 were anchored while the remaining genes could not be resolved due to a lack of marker/sequence information and cases where genes have been characterized but not genetically mapped (Youssef et al., 2025). A total of 469 QTL were collated (**Supplementary Table 1**), and 42 QRCs spread across 19 of the 21 chromosomes were identified (**Figure 1**). The B-sub genome had the highest number of clusters (20) followed by the A-sub genome (16) and the D-sub genome had the least number of clusters (6) with two chromosomes 5D and 6D lacking any clusters (**Supplementary Table 2**). QRCs and their co-location with known *Yr* genes were visualized on the wheat genome IWGSC RefSeq 2.1 using MapChart v2.32 (Voorrips, 2002) (**Figures 2A-C**). QRCs were also visualized as a circular heatmap using the R package Circlize (Gu et al., 2014) to illustrate QTL density within clusters and highlight corresponding regions across the A, B, and D sub-genomes (**Figure 1**). QRC intervals were plotted by physical position and colored according to the number of QTL within each region (**Figure 2c**). The number of resistance clusters identified in this study is lower than that reported in earlier analyses -for example, Rosewarne et al. (2013) identified 48 clusters derived from 140 stripe rust resistance QTL, while a meta-QTL analysis of 353 QTL reported 61 meta-QTL (MQTL) associated with stripe rust resistance (Jan et al., 2021). More recently, Tong et al. (2024) described 83 QTL-rich clusters (QRCs) based on 406 loci. These are four similar but different approaches to consolidate stripe rust resistance loci reported across independent studies. Here we did not conduct a formal meta-analysis because reported QTL did not provide sufficient information and vary considerably in confidence among studies. Historical mapping studies differ in population size, marker density, phenotyping design, number of environments, statistical thresholds and mapping resolution. Physical re-anchoring of loci using a 20 Mb confidence interval merged several previously designated **“**separate**”** clusters into fewer, broader genomic regions, thereby reducing the total number of clusters detected. Given the rapid advancement of genomic tools and the continued identification of resistance loci, future QTL discovery is expected to increase the density of established regions more often than total number of distinct clusters.

### 2.4 Sub-genome distribution and biological interpretation

The distribution of stripe rust resistance clusters across the wheat sub-genomes was inconsistent. The B sub-genome contained 47.61% of the total identified, consistent with a larger component of nucleotide-binding leucine-rich repeat (NLR) genes and enrichment for tandem duplications, features that promote the rapid evolution of new resistance specificities (Walkowiak et al., 2020) (**Figure 2B**; **Supplementary Table 2**). The A sub-genome contributed 38.10%, with the majority located in telomeric regions (**Figure 2A**; **Supplementary Table 2**). This distal enrichment aligns with genome-wide observations that almost half (approximately 46%) of genes in the A sub-genome; and an even higher proportion of resistance gene analogues, reside within the distal 20% of chromosome arms (International Wheat Genome Sequencing Consortium, 2018). In contrast, the D sub-genome accounted for only 14.29% (**Figure 2C**; **Supplementary Table 2**), with no clusters detected on chromosomes 5D and 6D, a pattern consistent with Jan et al. (2021) that also reported an absence of stripe rust resistance clusters on these chromosomes. The reduced representation of resistance loci in the D sub-genome likely reflects its lower genetic diversity, attributed to founder bottlenecks during the hexaploidization of wheat (Walkowiak et al., 2020). Together, this uneven distribution likely reflects the combined effects of wheat’s polyploid origin, sub-genome-specific diversity, recombination landscape, resistance-gene architecture, historical introgressions and long-term breeder selection for effective resistance haplotypes. In addition, a curated catalogue providing detailed descriptions of each QRC and *Yr* genes successfully projected on to the IWGSC RefSeq v2.1 is included (**Supplementary File 2**). This catalogue focuses on exploration of potential relationships among *Yr/QYr* loci as well as providing reported resistance type, source, pathotype information, pleiotropy and gene prediction where possible.

## 3 Discussion

### 3.1 WYR resistance in QRCs

In this review, catalogued *Yr*/*QYr* were systematically compiled from global literature sources. Where sufficient flanking or linked marker information was available, loci were physically projected onto the IWGSC RefSeq v2.1 Chinese Spring reference genome to standardize positional information across studies. This approach enabled the consolidation of overlapping reports and the identification of regions of co-localization.

From the analysis, 22 QRCs contained loci reported as ASR, with approximately half harboring catalogued *Yr* genes and the remainder comprising at least one QTL identified in seedling resistance assays. As several studies assessed resistance only at adult plant stages without seedling phenotyping, these cannot be confidently classified as ASR regions. Prominent ASR-rich clusters include chromosome 2A (1.64–56.84 Mb), which harbors *Yr17*, on the widely deployed 2NS/2AS translocation from *Aegilops ventricosa*; chromosome 2B (134.3–221.93 Mb) containing *Yr27* and *Yr31*; and chromosome 2B (622.75 – 725.09 Mb), which includes *Yr5*, *Yr7*, *Yr44, Yr43* and *Yr53*. These regions represent major reservoirs of seedling resistance and remain important targets for breeding and gene pyramiding.

Chromosome 1A is unique in lacking any catalogued *Yr* genes. However, two distinct telomeric clusters were identified, indicating the presence of uncharacterized resistance loci. While at least one locus within each cluster has been associated with seedling resistance, the underlying resistance type remains unconfirmed as most studies evaluated disease responses only at adult plant stages. A single cluster was defined on 1D (0.44–16.98 Mb). *Yr25* is the only reported ASR gene on chromosome 1D; physical placement is not possible as the gene has not been mapped. This QRC is therefore hypothesized to correspond to *Yr25*. Identifying candidate targets highlights the value of global projection.

Seven QRCs consisted mainly of catalogued APR genes, and four QRCs consisted of both ASR and APR genes. Large clusters occur in Chromosome 4B (525.99 – 588.18 Mb) that includes the APR genes *Yr62* and *Yr68*, and chromosome 6B (65.71 – 149.39 Mb) which contains the APR gene *Yr78* and the HTAPR gene *Yr36*. These large clusters are likely due to historical selections and repeated inclusion of these segments into elite germplasm because of the durability of APR genes (Ellis et al., 2014; Li et al., 2016; Dinglasan et al., 2022) through direct and indirect selections based on phenotype. Resistance loci are frequently organized in gene-rich regions (Richly et al., 2002; Young, 2000) where distinct ASR and APR loci can occur within the same physical interval. Therefore, these clusters are best interpreted as enriched resistance regions that may integrate loci with ASR and/or APR activity, rather than as discrete ASR-only regions.

As expected, large clusters were also associated with the pleiotropic genes *Yr18*, *Yr29* and *Yr30*, with 1B (667.28 – 685.02) cluster having over 20 loci reported to be likely or identical to *Yr29/Lr46*. In contrast, there was no cluster around *Yr46/Lr67* on chromosome 4D that was initially identified in a Pakistani landrace ‘PI 250413’ (Dyck and Samborski, 1979). Screening of CIMMYT’s breeding nursery between 2012 and 2013 with the *Yr46* marker revealed that none of its germplasm contained this gene (Moore et al., 2015). This was attributed to long-term selection of the semi-dwarfing gene *Rht-D1b* for short straw and high yield, discarding the resistant *Lr67* allele in the process (Hiebert et al., 2010; Moore et al., 2015). Alignment revealed a 4DL cluster at 439.53 - 478.05Mb about 30 Mb away from true *Yr46* positioned at 412.6 Mb. Although physically distinct, some of the loci in this cluster (*QYr.nmbu.4D*, (Lin et al., 2023) (*QYr.caas-4DL*, Bainong 64, (Ren et al., 2012) have been reported as allelic or closely linked to the pleiotropic *Yr46* locus.

Alignment further revealed 19 clusters with potential pleiotropic loci co-localizing with resistance to other pathogens including rusts (**Supplementary Table 3**). Interestingly, cluster 2A (689.67 – 763.64 Mb) and 7B (686.15 – 755.05 Mb) have three or more loci with leaf rust resistance. The 7B cluster also harbors *Yr6*, which was recently shown to be allelic to the powdery mildew gene, *Pm5*, in the Chinese cv. ‘Aikang 58’ (Wu et al., 2025). These QRCs showing consistent multi-disease effects represent priority targets for marker-assisted selection and pyramiding, as they enable simultaneous improvement of multiple resistance traits and are more likely to be durable.

### 3.2 Cloned genes validate QRCs as durable breeding targets

The cloning of multiple stripe rust resistance genes provides strong biological validation that many QRCs represent stable, high-value genomic regions that have been repeatedly selected for resistance across diverse germplasm and breeding programs (**Supplementary Table 4**). To date, 14 *Yr* genes have been cloned, with the majority localizing within defined QRCs. Notably, *Yr6* has been cloned twice using different approaches, *YrNAM* has been reported to be identical to *Yr10*, and *Yr5x* represents an allelic variant of *Yr5*. In addition, *YrKB* is yet to be catalogued and *YrU1* is temporarily designated. Several QRCs, most notably on chromosomes 1B, 2B, 5A, 6B, and 7B, harbor cloned genes encompassing diverse resistance mechanisms. The convergence of functionally distinct resistance genes within narrow physical intervals indicates that these regions act as long-term reservoirs of stripe rust resistance. The 7B QRC (686.15–755.05 Mb) exemplifies the functional complexity of genomic regions that have been repeatedly associated with stripe rust resistance, integrating the classical ASR *Yr6*, a paired NLR system with dual-function resistance, and the APR gene *YrKB*. The co-localization of ASR, adult-plant reistance, and polygenic resistance within a single physical interval highlights the functional diversity and breeding significance of this cluster. From our analysis, the ASR gene *Yr28* in chromosome 4D is the only cloned gene that does not occur within or neighboring a cluster. This likely reflects its origin as an introgressed resistance locus that has not been widely adopted due to potential linkage drag concerns (Zhang et al., 2019). A summary of candidate gene predictions within identified genomic regions is provided in **Supplementary Table 5**.

### 3.3 Genomic constraints and methodological challenges in defining stripe rust resistance clusters

Structural variation in wheat is strongly influenced by its polarized recombination landscape, with frequent recombination in distal chromosome regions and pericentromeric regions forming broad recombination deserts (Saintenac et al., 2009; Rowan et al., 2019). When resistance loci or associated markers intersect with low-recombination regions or structural variants, apparent positional displacement may occur, leading to inconsistencies in genetic and physical mapping. In addition, the repetitive nature of the genome and short sequences associated with markers can cause projection displacement. In such cases, markers may appear shifted within or even across chromosome arms or distal regions. This phenomenon has been observed for several stripe rust QTL, including *QYr.rcrrc.3B.1* identified on chromosome 3B in the IWWIP advanced line ‘130675’ (Tehseen et al., 2022). Genetic mapping placed this locus on the short arm of chromosome 3B, closely linked to the SNP marker 5971264 and likely corresponding to the seedling resistance gene *Yr4*, based on its avirulence to the PstS7 pathotype. Physical anchoring against the IWGSC RefSeq v2.1 assembly positioned the locus on the long arm (823.06–839.48 Mb). Similar inconsistencies were observed for *QYrlu.cau-2BS2* (genotype Luke), *QYrdr.wgp-6BL.1* (Druchamp), and *QYr.caas-4BS* (Dabaimai) (Guo et al., 2008; Hou et al., 2015; Wang et al., 2020), underscoring the widespread impact of genome architecture on QTL projection.

Anchoring resistance loci is further complicated by the frequent involvement of alien introgressions, which are largely absent from the Chinese Spring genome. The inferred physical position may depend on whether linked markers are derived from donor sequence or from associated wheat region. This can lead to inconsistent marker alignment, ambiguous QTL placement, and apparent cluster fragmentation across studies (Gill et al., 2011; Zhu et al., 2021). Accurate resolution of alien-derived resistance loci, therefore, increasingly depends on pan-genome assemblies, rye- and wild-relative–specific scaffolds, and long-read sequencing approaches capable of capturing structural variation and translocation breakpoints (Philippe et al., 2013; Yu et al., 2025; Zhang et al., 2025).

The integration of historical and modern datasets also reveals recurrent marker-related artefacts. In some cases, single markers have been considered to represent distinct resistance loci because they do not bind uniquely to a single genomic target due to paralogous priming or assay cross-amplification of similar sequences, as reported for *QYr.crc-2BS* (Thatcher) and *Yr7* (Rosa et al., 2019). Such marker behavior can generate spurious clustering signals and false-positive associations, particularly in diversity panels and meta-analyses (Millett and Bradeen, 2007; Poczai et al., 2013; Fang et al., 2020).

Historical limitations in mapping resolution have also resulted in redundant naming of genetically identical or near-identical loci, including *Yr24/Yr26, Yr34/Yr48* and *Yr4/YrRub* (Bansal et al., 2009; McIntosh et al., 2018; Qureshi et al., 2018). From a breeding and deployment perspective, this redundancy carries significant risk. Large-scale release of cultivars sharing identical resistance loci imposes strong and uniform selection pressure on Pst, accelerating the emergence of virulent races and increasing the probability of resistance breakdown. Collectively, these findings highlight the limitations of interpreting stripe rust resistance loci within isolated population-specific genetic maps and that apparent clustering, locus misplacement, and redundancy in stripe rust resistance mapping are largely driven by genome structure, recombination dynamics, alien introgressions, and marker limitations. These challenges highlight the need for structurally informed interpretation of QTL-rich clusters and reinforce the importance of high-resolution genomic resources, haplotype-based analysis, and pan-genome frameworks for robust resistance deployment in breeding programs.

## 4 Conclusion

This review consolidates four decades of stripe rust resistance research into a unified physical-genomic framework. By systematically compiling catalogued *Yr* genes and published QTL and projecting them onto a common physical map, this study demonstrates that interpreting resistance at the level of co-localizing genomic regions provides greater biological and practical clarity than considering individual loci in isolation. Many *Yr/QYr* co-locate within defined QRCs, which represent structured genomic regions enriched for both ASR and APR with diverse molecular architectures.

For breeding programs, this has immediate practical implications. Deployment strategies may benefit from prioritizing QRC-level haplotypes rather than individual named genes. Targeted stacking within validated clusters can enhance durability while minimizing the risk of inadvertently concentrating identical resistance sources under different designations. Conversely, the widespread release of cultivars carrying the same underlying haplotype increases uniform selection pressure on pathogen populations and accelerates the evolution of virulence.

The extensive catalogue generated in this study provides a consolidated, physically anchored resource detailing chromosomal positions, linked markers, and germplasm sources of *Yr/QYr* loci (**Supplementary Table 1**). This reference framework enables researchers and breeders to trace resistance back to original donor material, identify co-localized loci, and design informed stacking or haplotype-based selection strategies. Complementing this, **Supplementary File 2** provides a curated catalogue of *Yr/QYr* loci within each co-localizing cluster and details further information extracted from original QTL reports and potential relationships among loci. Beyond consolidating existing knowledge, this cluster-based framework also highlights genomic regions that remain underrepresented or unexplored in *Yr* research. By clarifying which intervals have been repeatedly characterized and which lack detailed investigation, this review provides researchers with a strategic roadmap to prioritize novel resistance regions for fine-mapping, gene discovery, and pre-breeding. Expanding efforts into these less-studied genomic segments will be essential for broadening the resistance base and reducing reliance on historically over-deployed loci.

Importantly, the genomic constraints and methodological challenges outlined in this review underscore why such consolidation is necessary. Genome structure, recombination dynamics, alien introgressions, and marker limitations can inflate the apparent diversity of reported loci and complicate comparisons across studies. By re-anchoring loci onto a common reference and defining QRCs, this work provides a structured framework that clarifies locus relationships and strengthens confidence in resistance deployment strategies.

## Supplementary material

Supplementary Table 1 Stripe rust resistance loci (*Yr*/*QYr*) compiled and physically anchored to the IWGSC RefSeq v2.1 Chinese Spring assembly, showing chromosome assignment, source genotype or population, linked marker(s), physical positions used to identify recurring genomic regions associated with stripe rust resistance and supporting reference.

Supplementary Table 2 Physical intervals of QRCs identified across the wheat genome from physically anchored *Yr/QYr*, showing chromosome assignment, cluster interval (Mb), and the number of reported loci contained within each cluster.

Supplementary Table 3 Co-localization of *Yr/QYr* loci with loci associated with other disease resistance traits within QRCs, highlighting genomic regions in which overlap may indicate pleiotropy or tight linkage, together with the source genotype or population, associated trait, and supporting reference.

Supplementary Table 4 Cloned *Yr* genes and their co-localization within QRCs, with resistance type, source species, protein class, cloning method and supporting reference.

Supplementary Table 5 Candidate genes predicted within QRCs associated with stripe rust resistance based on prior mapping studies and functional annotation, including source genotype or population, putative protein class, and supporting reference.

Supplementary File 2 Sub-genome resolved descriptions of stripe rust resistance clusters, detailing the individual *Yr* genes and QTL contained within each QRC and highlighting potential relationships among co-located loci.
