# Supplementary material for: QTL rich genomic clusters in wheat: a catalogue for resistance to Puccinia striiformis f. sp. tritici
Source: Front Plant Sci. 2026 Aug 4;17:1864074. doi: 10.3389/fpls.2026.1864074 (PMC13482860; doi:10.3389/fpls.2026.1864074)
Supplement: Supplementary file 1 [file SupplementaryFile2.docx]

Sub-genome descriptions of stripe rust resistance clusters

***Yr/QYr* Clusters on A Sub-Genome**

**Chromosome 1A**

**C1A.1 (5.95 – 44.52 Mb)**

This cluster on the telomeric end of the short arm of the chromosome contains one ASR locus, *QYr.nwafu-1AS*, that was identified in a diverse panel from various global regions and was effective against predominant Chinese Pst races, including CYR34 (Wu et al., 2020). Several QTL in this cluster confer resistance at the adult stage, and they include *QYrpd.swust-1AS*, identified in the winter wheat cultivar ‘Pindong 34’ (Zhou et al., 2022b); *QYrus.vt-1AS*, from the soft red winter wheat ‘USG 3555’ (Christopher et al., 2013) and *QYr.caas-1AS*, derived from the Chinese landrace ‘Dabaimai’ (Wang et al., 2020b). *QYr.AYH-1AS*, from a landrace ‘Anyuehong’ (Long et al., 2021); *QYr.sgi-1A* from a S. African spring wheat cultivar ‘Kariega’ (Prins et al., 2010) and *QYr.lrdc-1A.1* from ‘P2711’ (Farzand et al., 2022) are environment-dependent loci.

**C1A.2 (540.73 – 592.08 Mb)**

This cluster also occurs on the telomeric end of the long arm of the chromosome with an ASR locus, *QYr.nwafu-1AL*, (Wu et al., 2020) and several loci identified as conferring resistance at the adult stages. They include *QYrsv.swust-*1AL from a durum cv. ‘Svevo’ (Zhou et al., 2021), *QYr.spa-1A* from Canadian cv. ‘Stettler’ (Bokore et al., 2017), *QYr.lrdc-1A.2* from Canadian cv. ‘AAC Cameron’ (Farzand et al., 2022), *QYr.cim-1AL* from a susceptible CIMMYT line ‘Apav#1’ (Liu et al., 2022a), *QYr.caas-1AL* from a German cv. ‘Naxos’ (Ren et al., 2012b), and *QYrPI197734.wgp-1A* from a Swedish spring cv. ‘PI 197734’(Liu et al., 2020a).

**Chromosome 2A**

**C2A.1 (1.64 – 56.84 Mb)**

In the distal region, the widely used Aegilops ventricosa-derived 2NS/2AS translocation harbours the tightly linked gene cluster *Yr17*/*Sr38*/*Lr37*/*Pm4* complex. The French wheat line ‘VPM1’, derived from *A. ventricosa,* was originally selected for its resistance to eyespot (strawbreaker foot rot) but exhibited strong seedling resistance to rusts (Bariana and McIntosh, 1993; McIntosh et al., 1995). Monosomic and telocentric analyses confirmed a tightly linked cluster of single dominant genes conferring seedling ASR with no recombination between *Sr38* and *Yr17*, and 0.5 ± 0.5% recombination between *Sr38*/*Yr17* and *Lr37* (Bariana and McIntosh, 1993). Several QTL in this cluster are likely the same as *Yr17,* including *QYr.lrdc-2A.1* (P2711) (Farzand et al., 2022) and the pleiotropic locus *QYr.hebau-2AS/QLr.hebau-2AS* (SW 8588) (Zhang et al., 2019b) confirmed using the PCR marker VENTRIUP/LN2, specific for the 2NS/2AS translocation; *QYr.orst-2AS* (Tubbs) supported by its linkage to the marker *cfd36* and pedigree (Madsen carries the *Yr17* gene) (Vazquez et al., 2015b).

This translocation also harbours non-race-specific HTAP loci. *YrM1225* was identified in mutant line ‘M1225’ (from AvSYr17NIL) and was effective against *Yr17*- virulent races at adult stages under high temperatures (Li et al., 2023). The same study estimated the 2N^v^S translocated segment to be 20.6 Mb based on ten co-segregating markers, based on IWGSC RefSeq v1.0 (Li et al., 2023). A similar study reported a race-specific APR locus *QYr.cim-2AS/QLr.cim-2AS* in the CIMMYT wheat line ‘Mucuy’, in addition to an ASR locus *YrMu,* and they co-segregated with *Yr17*-linked markers WGGB156 and WGGB159 (Liu et al., 2022a).

An ASR gene, *Yr69*, was identified in an introgression line ‘CH7086’ from *Thinopyrum ponticum*-derived partial amphiploid Xiaoyan 7430. It differs from *Yr17* based on resistance specificity. Allelism tests revealed that though they may be linked, they are not allelic (Hou et al., 2016). An ASR locus, *QYr.sicau-2AS* (Shumai126), likely corresponds to *Yr69* based on identical genotypes produced with a *Yr69*-carrying line using KASP markers and overlapping physical intervals (Wang et al., 2022). Candidate gene prediction within a 2.75 Mb region based on RNA-seq expression data identified TraesCS2A02G016200 encoding an NB-ARC protein (Wang et al., 2022).

An ASR gene, *Yr56 (QYr.sun-2A)*, was identified in the durum wheat cv. ‘Wollaroi’ against Australian pathotypes (134 E16A+, 134 E16A+Yr17+, 134 E16A+J+T+) (Bansal et al., 2013; Wang et al., 2021b). An APR QTL, *QYr.uga-2AS (YrR61)*, was identified in a winter cv. ‘Pioneer 26R61’ and was confirmed to be different from *Yr17* using the VENTRIUP/LN2 marker (Hao et al., 2011). *QYrva.vt-2AS* from the winter cv. ‘VA00W-38’ was reported to be similar to *YrR61* due to marker overlap (Christopher et al., 2013). Similarly, *QYr.ufs-2A* from ‘Yr16DH70’ (Cappelle-Desprez-derived line) was reported to be linked to *YrR61* (Agenbag et al., 2012).

Candidate gene prediction within regions of *Qyr.gaas-2A* (Guixie3) (Cheng et al., 2022) and *QYr.hbaas-2AS* (Jia et al., 2020) identified NBS-LRR genes. Three WRKY transcription factors were also identified for *Qyr.gaas-2A* (Cheng et al., 2022). Gene prediction in the *QYr.GX-2AS* (Gaoxianguangtoumai (GX)) region identified eight genes differing from known yellow rust genes (Wang et al., 2021b). They include RING/U-box, ascorbate peroxidase, glycosyltransferases, and F-box family proteins, potentially involved in disease resistance (Wang et al., 2021b).

**C2A.2 (689.67 – 763.64 Mb)**

The ASR gene *Yr1* likely originated in the cv. ‘Chinese 166’, which serves as a reference stock (Lupton and Macer, 1962; Macer, 1966; McIntosh et al., 1995) and determined to be located on the long arm of the chromosome (Macer, 1966; Bariana and McIntosh, 1993). The ASR gene *Yr32* (*YrCV*) was originally identified in the cv. ‘Carstens V’ (Eriksen et al., 2004). Both cv. Chinese 166 and Carstens V have been used in stripe rust differential sets (Johnson et al., 1972; Eriksen et al., 2004).

An APR gene, *Yr86* (*QYryz.caas-2AL*), was identified in the Chinese cultivar ‘Zhongmai 895’ and was effective against prevalent Chinese *Pst* pathotypes (Zhu et al., 2023). Candidate gene prediction within the region identified NB-ARC, TIR-NBS-LRR (TNL) and protein kinase domains (Zhu et al., 2023; Cao et al., 2024). Similarly, gene prediction within the region of *QYr.rcrrc-2A* (130675 – IWWIP) detected NB-ARC and protein kinase domains (Tehseen et al., 2022).

Potentially pleiotropic or closely linked loci in this cluster include *QYr.ifa-2AL/QLr.ifa-2AL* (Capo) (Buerstmayr et al., 2014), *QYr-2AL.2/QLr-2AL.2* (Zhang et al., 2021) and *QYr.cim-2AL/QLr.cim-2AL* (KU3067) (Zhang et al., 2022).

**Chromosome 3A**

**C3A.1 (7.0 – 25.98 Mb)**

The ASR gene *Yr76 (YrTye)* identified in the cv. ‘Tyee’ was originally mapped in chromosome 6D by monosomic analysis and later to 3AS (Chen, 1995; Xiang et al., 2016). Another ASR QTL, *Yrq2*, was identified in a Chinese slow-rusting cv. ‘Xichang 76-9’ (Cao et al., 2012) .

All the QTL that occur in this cluster were identified in field-based studies, and they include *QYr.nmbu.3A.1* (Lin et al., 2023), *QYr.cim-3AS* (Mucuy) (Liu et al., 2022a), *QYr.ifa-3AS* (Arina) (Buerstmayr et al., 2014) and *QYrsv.swust-3AS* (Svevo) (Zhou et al., 2021).

**C3A.2 (50.59 – 54.76 Mb)**

This cluster includes QTL conferring resistance at the adult stage, including *QYr.hbau-3AS* (Mianyang351-15) (Gebrewahid et al., 2020b), *QYr.dms-3A* (CDC Go) (Zou et al., 2017) and *QYrne.vt-3A* (Neuse) (Christopher et al., 2012).

**C3A.3 (715.13 – 740.73 Mb)**

This cluster includes an ASR QTL *QYr.nwafu-3AL*, and field-detected loci *QYr-3AL* (Zhang et al., 2021), *QYrpd.swust-3AL.2* and *QYrpd.swust-3AL.1* (Pindong 34) (Zhou et al., 2022b) and qNV.Yr-3A.5 (Jambuthenne et al., 2022). It also includes a pleiotropic locus *QYr.hebau-3AL/QLr.hebau-3AL* identified in the cv. ‘Fuyu 3’ (Gebrewahid et al., 2020a).

**Chromosome 4A**

**C4A.1 (670.31 – 745.22 Mb)**

Two catalogued ASR genes occur in this cluster. The gene *Yr51* was identified in the Pakistan landrace ‘AUS27858’ and was effective against six Australian Pst pathotypes, including 134 E16A+Yr17+Yr27+ (Randhawa et al., 2014). *Yr60* was identified in the wheat line ‘Almop’, conferring moderate resistance at both seedling and adult-plant stages (Herrera-Foessel et al., 2015).

Two other ASR loci occur in this cluster, and they include *QYr.nwafu-4AL* (Wu et al., 2020) and *QYrel.wgp-4AL* (Eltan) (Liu et al., 2019a).

The cluster also includes several field-detected loci, including *qNV.Yr-4A.1* and *qNV.Yr-4A.2* (Jambuthenne et al., 2022), *QYrsv.swust-4AL* (Svevo) (Zhou et al., 2021), *QYr.orr-4AL* (Stephens) (Vazquez et al., 2012), *QYr.sgi-4A.1* (Kariega) (Agenbag et al., 2014; Pretorius et al., 2016) and *QYrcen.nwafu-4AL* (Centrum) (Mu et al., 2019).

**Chromosome 5A**

**C5A.1 (6.76 – 31.83 Mb)**

This cluster contains three QTL, an ASR *QYr.nwafu-5AS* (Wu et al., 2020) and two loci that confer resistance at the adult stage; *Qyr.gaas.5A.1* (Guixie3) (Cheng et al., 2022) and *QYr-5AS* (Zhang et al., 2021).

**C5A.2 (452.27 – 464.32 Mb)**

This cluster also has three loci, a potential pleiotropic locus *QYr.hebau-5AL/QLr.hebau-5AL* (Fuyu 3) (Gebrewahid et al., 2020a) and two loci that confer resistance at the adult stage; *Qyr.gaas.5A* (Mian 96–5) (Cheng et al., 2022) and *QYr.ifa-5A* (Capo) (Buerstmayr et al., 2014).

**C5A.3 (489.1 – 549.28 Mb)**

*YrU1* is an ASR gene identified in a diploid wheat progenitor, *Triticum urartu* accession ‘PI428309’, conferring resistance to predominant Chinese races, including CYR33 (Wang et al., 2020a). Map-based cloning of *YrU1* revealed that it encodes a unique CC-NBS-LRR protein with an N-terminal ankyrin-repeat (ANK) domain and C-terminal WRKY domain (Wang et al., 2020a). An ASR QTL *QYr.nwafu-5AL* identified in a GWAS study was also effective against Chinese races, including CYR33 and CYR34 (Wu et al., 2020).

This cluster also includes QTL conferring resistance at the adult stage; *QYr.nmbu.5A.1* and *QYr.nmbu.5A.2* (Lin et al., 2023), *QYrsv.swust-5AL.1* (Svevo) (Zhou et al., 2021) and *qNV.Yr-5A* (Jambuthenne et al., 2022).

**C5A.4 (576.17 – 610.6 Mb)**

This cluster consists of a potentially pleiotropic locus *QYr.hebau-5AL/QLr.hebau-5AL* (SW 8588) (Zhang et al., 2019b) and QTL that confer resistance at the adult stage; *Qyr.gaas.5A.2* (M96-5) (Cheng et al., 2022), *QYr.lrdc-5A* (P2711) (Farzand et al., 2022) and *QYrsv.swust-5AL.2* (Svevo) (Zhou et al., 2021).

The QTL *QYrsk.wgp-5AL* identified in the winter wheat cv. ‘Skiles’ may not be a true resistance QTL but rather an artifact associated with the vernalization gene *Vrn-A1* (Liu et al., 2019b). Its proximity to *Vrn-A1* and association with growth habit differences suggest it may reflect vernalization effects rather than true stripe rust resistance (Liu et al., 2019b).

**C5A.5 (641.46 – 710.84 Mb)**

The ASR gene *Yr34 (YrWA)* was identified in an Australian breeding line ‘WAWHT2046’, effective against Pst pathotype 134 E16A+, and is linked to the awn inhibitor gene *B1* (Bariana et al., 2006). The APR gene *Yr48* was identified in a CIMMYT synthetic derivative cv. ‘PI610750’ and it is completely linked to the vernalization locus *Vrn-A2* (Lowe et al., 2011). A subsequent study established that *Yr48* is a synonym of *Yr34*, based on identical haplotypes, allelism tests, and similar partial HTAP responses against pre-2002 (110 E143A+) and post-2002 (134 E16A+Yr17+Yr27+) pathotypes (Qureshi et al., 2018; Chen et al., 2021). *Yr34/Yr48* was shown to be located within a distal segment of *Triticum monococcum* chromosome 5AmL, translocated to chromosome 5AL in polyploid wheat (Chen et al., 2021). Candidate gene prediction within a 9.5Mb region of *Yr34/Yr48* revealed that it may encode receptor-like protein kinase (RLK) genes (Chen et al., 2021).

The QTL *QYr.rcrrc-5A* (130675) (Tehseen et al., 2022), *QYrdr.wgp-5AL* (Druchamp) (Hou et al., 2015) and *QYr.crc-5AL* (Toropi 6.4) (Rosa et al., 2019) were reported to be likely similar to the locus *Yr34/Yr48*.

Other QTL in this cluster include a potentially pleiotropic locus *QYr.spa-5A* (Lillian) that co-locates with a powdery mildew resistance QTL *QPm.spa-5A* (Bokore et al., 2017), and *QYr.caas-5AL.2* (Shanghai 3/Catbird), which confers resistance at the adult stage (Ren et al., 2012b).

**Chromosome 6A**

**C6A.1 (8.09 – 35.8 Mb)**

The APR gene *Yr81 (YrAW5)* was identified in a Chinese landrace from the Watkins Collection ‘Aus27430’, and it exhibited moderate resistance to prevalent Australian Pst pathotypes (Gessese et al., 2019). *QYr.nwafu-6AS* identified in a GWAS study co-localised with *Yr81* and had the positive allele using the KASP_3077 (*Yr81*) marker (Wu et al., 2020).

Other QTL in this cluster confer resistance at the adult stages and they include; *QYr.uga-6AS* (Pioneer 26R61) (Hao et al., 2011), *QYrex.wgp-6AS* (Express) (Lin and Chen, 2008), *QYrel.wgp-6AS* (Eltan) (Liu et al., 2019a), and *QYr.sicau-6AS* (Shumai 126) (Wang et al., 2022).

**C6A.2 (612.2 – 622.59 Mb)**

This cluster consists mainly of loci conferring resistance at the adult stage, and they include *YrJ44* (Jimai 44) (Liu et al., 2023), *Qyr.gaas.6A* (Guixie 3) (Cheng et al., 2022), *QYrtb.orz-6AL* (Einstein) (Vazquez et al., 2015a), *QYr.nmbu.6A* (Lin et al., 2023), *qNV.Yr-6A* (Jambuthenne et al., 2022).

Candidate gene prediction within the intervals of *Qyr.gaas.6A* (Guixie 3) and *QYr.nmbu.6A* revealed that they may potentially encode TIR-NBS-LRR and NBS-LRR genes, respectively (Cheng et al., 2022; Lin et al., 2023).

**Chromosome 7A**

***Yr61***

The ASR gene *Yr61* identified in a winter cv. ‘Pindong 34’ was effective against prevalent Chinese Pst pathotypes, including CYR32 and CYR33 (Zhou et al., 2014a).

**C7A.1 (717.83 – 734.38 Mb)**

*Yr75 (YrAxe)* is an APR gene that was identified in the Australian cv. ‘Axe’ conferring a resistant to moderately resistant response (RMR) against pathotype 134E16A+Yr17+Yr27+ (Kanwal et al., 2021). QTL in this cluster confer resistance at the adult stage and include qNV.Yr-7A.2 (Jambuthenne et al., 2022), *QYr-7AL.2* (Zhang et al., 2021) and *QYr.nmbu.7A* (Lin et al., 2023).

***Yr/QYr* Clusters on B Sub-Genome**

**Chromosome 1B**

**C1B.1 (1.52 – 17.54 Mb)**

The ASR gene, *Yr10*, was originally identified in a Turkish winter landrace ‘PI 178383’ and was found to be associated with a gene for brown glume colour (Lewellen et al., 1967; Metzger and Silbaugh, 1970). It was initially deployed in Avocet S + *Yr10* NILs and the cv. Moro, which has been widely used globally (Ni et al., 2023; Dibley et al., 2024). *Yr10* was initially identified as Yr10cg and encoded a putative CC-NBS-LRR gene, but has recently been shown to encode a non-canonical resistance protein with NAM (No Apical Meristem) and ZnF-BED (BED Zinc Finger) domains, distinct from *Yr10cg*’s CC-NBS-LRR structure (Dibley et al., 2024). An ASR QTL, *QYr.nwafu-1BS.1*, was identified in a GWAS and confirmed as *Yr10* using KASP markers (Wu et al., 2020).

An ASR gene, *Yr84*, was identified in a wild emmer wheat ‘PI 487260’ using BSA-Seq and was shown to exhibit incomplete dominance. They confirmed it to be different from *Yr10* through race-specificity tests (Klymiuk et al., 2022; Klymiuk et al., 2025). Cloning and functional validation demonstrated *Yr84* encodes two co-dependent NLRs, CC-NB-ARC-LRR (CNL) and NB-ARC-LRR (NL) genes (Klymiuk et al., 2025).

Other QTL conferring resistance at the adult stage include *QYr.hbaas-1BS* (Jia et al., 2020) and *QYr.cim-1BS* (KU3067) (Zhang et al., 2022). Candidate gene prediction with the region of *QYr.hbaas-1BS* identified three NBS-LRR genes and two receptor-like kinase (RLK) genes, with the NBS-LRR candidates showing high post-inoculation expression, indicating strong candidacy (Jia et al., 2020).

***Yr85***

The ASR gene, *Yr85*, originally identified in the club wheat ‘Tres’, was previously named *YrTr1* and was found to occur in chromosome 6D (Chen and Line, 1992; Feng et al., 2023). Using a near-isogenic line (AvSYrTr1NIL), nulli-tetrasomic and deletion line analysis confirmed its location in 1B and was shown to be different from *Yr10* using race specificity tests (Feng et al., 2023).

***Yr15***

The ASR gene, *Yr15*, was originally derived from *T. dicoccoides* ‘G25’ and proved to be highly resistant in multiple environments against the predominant races in the 1970’s (Gerechter-Amital and Grama, 1974; Gerechter-Amitai et al., 1989). Cytogenic analysis and linkage mapping placed *Yr15* on the short arm of 1B and was shown to be different from *Yr10* using race specificity tests (McIntosh et al., 1996). Map-based cloning and functional validation showed that *Yr15* encodes a broad-spectrum tandem kinase-pseudokinase (TKP) gene (Klymiuk et al., 2018).

***Yr24/Yr26***

The ASR genes *Yr24* and *Yr26* are examples of single resistance genes identified from multiple sources. *Yr24* was first mapped to the centromeric region of 1BS in a synthetic wheat derivative from durum accession ‘K733’ crossed with *A. tauschii* against an early Australian Pst pathotype (150 E16 A+) and was found to be closely linked to *Yr15* (McIntosh and Lagudah, 2008; McIntosh et al., 2018). *Yr26* was initially identified in China in materials derived from *Dasypyrum villosum*, initially thought to be linked to the powdery mildew resistance *Pm21* but later confirmed to be on chromosome 1B, independent of *Pm21* (Yildirim et al., 2000; McIntosh et al., 2018). A recessive gene, *YrGn22*, was identified in another derivative of *D. villosum,* ‘Guinong 22’, mapped to the centromeric region and was later shown to be linked to, or possibly similar to, *Yr26* (Zhang et al., 1998; Han et al., 2015; McIntosh et al., 2018). *YrCH42* was identified in the Chinese cv. ‘Chuanmai 42’ and located near the centromere. Tests for allelism with *Yr24* and *Yr26* indicated that they are likely the same gene or allelic at the same locus based on seedling test’s reaction patterns (Li et al., 2006).

***Yr64* and *Yr65***

The ASR genes *Yr64* and *Yr65* were identified in Ethiopian durum cv. ‘PI 331260’ and ‘PI 480016’ respectively in the U.S and they were mapped to 1BS. Tests for allelism and marker distances showed that they were distinct from each other (Cheng et al., 2014). The same study also demonstrated that the two genes were different from *Y24/Yr26* through race specificity tests and from *Yr10* through flanking marker distances (Cheng et al., 2014).

**C1B.2 - (326.00 – 353.60 Mb)**

This cluster includes an ASR QTL, *QYr.nwafu-1BL,* identified in a GWAS study and colocalises with the ASR gene *Yr24/Yr26* using KASP markers (Wu et al., 2020). *QYrdr.wgp-1BL.1* was identified in the cultivar ‘Druchamp’, conferring durable HTAP resistance (Hou et al., 2015). *QYr.caas*-*1BL.1RS* was identified from a CIMMYT cv. ‘Shanghai 3/Catbird (SHA3/CBRD)’ and is located near the centromere, co-locating with a powdery mildew QTL and linked to the marker Xiag95 (Ren et al., 2012b). They concluded that it was possibly a novel locus from the *Yr9* locus based on race specificity and chromosome location (Ren et al., 2012b).

**C1B.3 - (667.28 – 685.02 Mb)**

This cluster primarily represents the pleiotropic slow-rusting gene *Yr29* (*Lr46/Yr29/Sr58/Pm39/Ltn2*), conferring durable, race-non-specific APR against stripe rust, leaf rust, stem rust, and powdery mildew. It is also associated with leaf tip necrosis (LTN), a morphological trait used as a visual marker and occurs in numerous different backgrounds (Herrera-Foessel et al., 2011; Zhou et al., 2021; Tong et al., 2024).

*Lr46* was initially identified in the Mexican CIMMYT-derived cv. ‘Pavon 76’ and was later associated with *Yr29* (Singh et al., 1998; William et al., 2003). It was also identified in the CIMMYT cultivar ‘Attila (Pseudo – PBW343)’ and was shown to be linked to a small plant defence gene family encoding β-1 proteasome subunits (Rosewarne et al., 2006; Rosewarne et al., 2008). Molecular markers used in the identification of *Yr46/Yr29* include *csLV46G22* (Gebrewahid et al., 2020a; Liu et al., 2020a; Zhang et al., 2021; Bobrowska et al., 2022; Huang et al., 2022), *Xwmc44* (Roder et al., 1998; Hou et al., 2015) and *Xgwm259* (Lowe et al., 2011; Hou et al., 2015). Bobrowska et al. (2022) reported that csLV46G22 and Xwmc44 are the most reliable markers for identifying *Lr46/Yr29*.

Loci that occur in this cluster (likely *Yr29*) include *QYr.spa-1B* (Vesper) (Bokore et al., 2017), *Qyr.gaas.1B* and *Qyr.gaas.1B.1* (Guixie 3) (Cheng et al., 2022), *QYrPI181410.wgp-1BL* (PI 181410) (Liu et al., 2020a), *QYr.crc-1BL* (Toropi 6.4) (Rosa et al., 2019), *QYr.spa-1B* (Carberry) (Bokore et al., 2017), *QYrsn.nwafu-1BL* (Shaannong 33) (Huang et al., 2021), *QYr.rcrrc.1B* (IWWIP 130675) (Tehseen et al., 2022), *QYr.sicau-1BL* (Shumai 126) (Wang et al., 2022), *QYr.cim-1BL* (Mucuy) (Liu et al., 2022a), *QYrdr.wgp-1BL.2* (Druchamp) (Hou et al., 2015), *QYrsk.wgp-1BL* (Skiles) (Liu et al., 2019b), *QYr.cim-1BL* (Kenya Kongoni) (Calvo-Salazar et al., 2015), *QYr.hbau-1BL* (Fuyu 3) (Gebrewahid et al., 2020a), *QYrCW357-1BL* (Changwu357-9) (Huang et al., 2022), *QYr.nwafu-1BL.3* (Jimai 44) (Liu et al., 2023), *QYr.hebau-1BL* (SW 8588) (Zhang et al., 2019b), QYr-1BL.2 (Zhang et al., 2021), *QYr.sdau-1BL* (Tutoumai (TTM)) (Pang et al., 2022), *QYrcw.nwafu-1BL* (Chakwal 86 (CW86)) (Zeng et al., 2019a) and *QYr.cim-1BL* (Francolin#1) (Pretorius et al., 2016).

Candidate prediction within a 500kb of this region has identified genes that include those that encode NB-ARC domains (involved in pathogen recognition), protein kinase domains (key in signalling pathways), and leucine-rich repeat (LRR) proteins (Tehseen et al., 2022). Notably, Pang et al. (2022) identified a receptor-like protein kinase (TraesCS1B01G454600) located within a 332kb region of *Yr29*.

**Chromosome 2B**

**C2B.1 (34.06 – 74.85 Mb)**

This cluster includes an ASR locus, *QYr.nwafu-2BS.1*, identified in China (Wu et al., 2020), and several QTL conferring adult-stage resistance. The QTL include *QYr.spa-2B.1* from a Canadian cv ‘Carberry’ (Singh et al., 2013; Singh et al., 2014). It colocalises with a stem rust resistance QTL (*QSr.spa-2B*) and a pseudo-black chaff (PBC) QTL (*QPbc.spa-2B*) in the same population (Singh et al., 2013).

*QYr.sgi-2B.1* (Kariega) (Ramburan et al., 2004; Prins et al., 2010) may correspond to *QYr.lrdc-2B* (AAC Cameron) (Farzand et al., 2022), as the flanking SSR marker Xbarc200 of *QYr.sgi-2B.1* is less than 4 cM from the SNP marker Tdurum_contig54704_176, and physical positions overlap (Farzand et al., 2022). *QYrid.ui-2B.1* (IDO444) (Chen et al., 2011) and *QYrlu.cau-2BS1* (Luke) (Guo et al., 2008) are HTAP loci identified under high temperature.

**C2B.2 (134.25 – 221.93 Mb)**

This cluster contains two catalogued genes and several QTL. The ASR gene, *Yr27*, previously known as *YrSk* or the ‘Selkirk’ gene (McIntosh et al., 1995), is present in many wheat cultivars, including Opata 85 and Ciano 79 and was widely distributed by CIMMYT (McDonald et al., 2004). Monosomic analysis and teleocentric mapping showed that it is closely linked to the leaf rust resistance gene *Lr13* in repulsion and *Lr23* (McDonald et al., 2004). A South African cultivar, Kariega, has consistently shown good resistance to stripe rust since its release in 1993, and this resistance is attributed to three loci, *QYr.sgi*-*2B.1*, *QYr.sgi-4A.1*, as well as the pleiotropic locus *Lr34/Yr18/Sr57* (Ramburan et al., 2004; Prins et al., 2010; Agenbag et al., 2014; Pretorius et al., 2016). Long read sequencing of the cv ‘Kariega’ revealed that *QYr.sgi-2B.1* locus is the same as the race-specific gene *Yr27,* and it encodes a nucleotide-binding leucine-rich repeat (NB-LRR) immune receptor. The same study reported allelism between *Yr27* and *Lr13,* with their protein products sharing 97.3% sequence identity (Athiyannan et al., 2022).

*Yr31* is a race-specific gene first reported in the CIMMYT cv. ‘Pastor’ (Singh et al., 2003). It is found in many cultivars and is located in the same region as leaf rust resistance genes *Lr13* and *Lr23* (Rosewarne et al., 2012; Rosewarne et al., 2013; Yang et al., 2013).

*YrF* (*QYr.cim*-*2BS*) is a seedling resistance gene identified in the CIMMYT cv. Francolin#1 that confers intermediate seedling and moderate APR in Chinese and Mexican environments (Lan et al., 2014a; Lan et al., 2014b; Pretorius et al., 2016).

A pleiotropic locus *QYr.hebau-2BS/QLr.hebau-2BS* was identified in the cv. ‘Fuyu3’ in multiple environments (Gebrewahid et al., 2020a). Several other QTL occur in this cluster, including *QYrlo.wpg-2BS* (Louise) (Carter et al., 2009), *Qyryac.nwafu-2BS* (Yaco “S”) (Li et al., 2017), *QYr.ucw-2BS* (UC1110) (Lowe et al., 2011), *QYrlov.nwafu-2BS* (P10057) (Wu et al., 2017), *QYr.spa-2B* (AC Cadillac) (Bokore et al., 2017) and *QYrhm.nwafu-2BC* (Humai 15) (Yuan et al., 2018). Wu et al. (2017) noted that *QYrlov.nwafu-2BS* may be allelic to other QTL in CIMMYT-derived cv., such as Kariega, Louise, Luke and Opata 85 mapped in the same region.

Candidate gene prediction with the *QYrhm.nwafu-2BC* region identified a protein kinase with ATP-binding activity and a disease resistance protein with NB-ARC motif, potentially contributing to resistance signalling and hypersensitive response (Yuan et al., 2018).

***Yr41***

The ASR gene, *YrCN19*, was identified in Chinese lines ‘AIM5 and AIM6’ and cv ‘Chuan-nong19 (CN19)’ using SSR markers (Xgwm410 (0.3 cM)) and showed high resistance (IT 0) to prevalent races CYR 30-32 (Luo et al., 2005). Allelism tests were conducted to confirm the novelty of *YrCN19* in CN19, and it was renamed *Yr41* (Luo et al., 2008). Jiang et al. (2020) demonstrated the effectiveness of Yr41 in ‘AIM5 and AIM6’ against the predominant races in China, including CYR 34.

**C2B.3 (622.75 – 725.09 Mb)**

The ASR gene, *Yr5*, derived from *T. spelta album*, was first reported and localised by Macer using monosomic analysis (Macer, 1966; Luo et al., 2005). It has been used extensively and continues to be used, and several closely linked markers have been developed (Chen et al., 2003; Yan et al., 2003; Sun et al., 2008; Naruoka et al., 2016; Marchal et al., 2018). The ASR gene, *Yr7*, was initially identified in the cv ‘Lee’ and mapped using monosomic analysis (Macer, 1972; Luo et al., 2009). It was widely deployed, and its significance was evident during the 1980’s pandemic (de Vallavieille-Pope et al., 1990). *YrSP* (*Yr5b*) was initially identified from the cv ‘Spalding Prolific’(McIntosh et al., 1995) and is still used in some countries against specific races (McIntosh et al., 1995; Calonnec et al., 2002). For a long time, it was thought that *Yr5* and *Yr7* are allelic variants and closely linked to *YrSP* (Marchal et al., 2018; McIntosh, 2024). Gene cloning using MutRenSeq revealed that *YrSP* is a truncated *Yr5* (99.8% identity) and thus suggests allelism, whereas *Yr7* is distinct (77.9% identity to *Yr5*) and possibly a paralog or divergent allele (Marchal et al., 2018). All three genes encode BED-NLRs with a BED domain, an NB-ARC domain, and variable LRRs, which are distinct from typical NLRs (Marchal et al., 2018). *Yr5x* is a newly identified allele at the *Yr5* locus that confers resistance to Pst race TSA-V5 and other Chinese races, including CYR32. Gene cloning has revealed that it encodes an NLR with BED, NBS, and LRR domains (Wu et al., 2025).

The ASR gene, *Yr43*, was identified in the spring wheat cultivar ‘IDO377s’ and confirmed using ditelosomic and nulli-tetrasomic lines. It was effective against PST-43 and PST-45 in the greenhouse, but its resistance was overcome after 2005 by PST-116 and PST-127 (Cheng and Chen, 2010). The ASR gene, *Yr44 (YrZak),* was identified in the cv. ‘Zak’ and tests for allelism confirmed it to be different from *Yr43* and *Yr5* (Cheng and Chen, 2010). The ASR gene, *Yr53*, was identified in an Ethiopian durum wheat ‘PI 480148’ (IT 2) and was consistently effective for several years against all US Pst races. Allelism tests showed it to be different from *Yr5,* and its location was confirmed using deletion and nulli-tetrasomic lines (Xu et al., 2013).

An ASR locus, *QYr.inra-2BL,* identified in the cv. ‘Apache’ was reported as similar to *Yr7* based on race specificity tests (Paillard et al., 2012). *QYrns.orz-2BL* detected in the cv. ‘NSA-98-0995’ in multiple environments in the U.S. may correspond to *Yr5* (Vazquez et al., 2015a). Its marker *wPt-0950* also aligns with a Cephalosporium stripe QTL (Vazquez et al., 2015a).

The QTL *QYr.caas-2BL* identified in the German cv. ‘Naxos’ co-locates with a powdery mildew and Fusarium head blight resistance QTL, suggesting potential pleiotropy or tight linkage (Ren et al., 2012b). Candidate gene prediction within *QYrSM155.1* (Shaanmai 155) region identified three high-confidence genes encoding pathogenesis-related proteins (Zhou et al., 2022a).

Other QTL that occur in this cluster include *QYr.nafu-2BL* (P9897) (Zhou et al., 2015), *QYrsnb.nwafu-2BL* (Snb“S”), *QYr.niab-2B* (Claire) (Powell et al., 2013), *QYraq.cau-2BL* (Aquileja) (Guo et al., 2008), *QYrqin.nwafu-2BL* (Qinnong 142) (Zeng et al., 2019b) and *QYr.nmbu-2B.1* (Lin et al., 2023).

**C2B.4 (776.34 – 809.79 Mb)**

An ASR gene *Yr72(YrAW4)* was identified in two landraces from the Watkins Collection, ‘AUS27506’ and ‘AUS27894’, and it was effective against the Australian Pst isolate 134 E16A+17+27+ (Chhetri et al., 2023). *QYr.nwafu-2BL* identified in a GWAS study against prevalent Chinese *Pst* races was reported to be likely *Yr72* (Wu et al., 2020).

Other loci in this cluster include an ASR locus *QYrpd.swust-2BL.4* and field-detected loci *QYrpd.swust-2BL.1*, *2BL.2* and *2BL.3* from the cv. ‘Pingdong 34’ (Zhou et al., 2014a) and *QYr.ifa-2BL* from the cv. ‘Capo’, which co-locates with a leaf rust QTL *QLr.ifa-2BL* (Buerstmayr et al., 2014).

**Chromosome 3B**

**C3B.1 (2.51 – 21.45 Mb)**

The slow rusting APR gene, *Yr30*, was first identified in the bread wheat cultivar ‘Opata 85’ in Mexico using RFLP markers (Singh et al., 2000). It was shown to be closely linked to the leaf rust resistance gene Lr27 and the stem rust resistance gene *Sr2,* suggesting pleiotropy (Singh et al., 2000). The gene complex is also linked to a powdery mildew resistance gene, Pm70, and the morphological trait, pseudo-black chaff (PBC) (McIntosh et al., 1995; Singh et al., 2000; Wang et al., 2024). Expression of PBC varies with genetic backgrounds and environmental conditions, making it a useful but variable marker for breeding. It is thought to be associated with cell death responses and is potentially linked to the lesion mimic locus, which may enhance resistance through programmed cell death mechanisms (Tabe et al., 2019). Wang et al. (2024) identified five candidate genes for the *Yr30* region based on functional annotation, expression differences (via RNA-seq), and sequence polymorphisms between cultivars with and without *Yr30*. The annotation encoded glycosyltransferase, dephospho-CoA kinase, murein hydrolase activator NlpD, glycerol-3-phosphate acyltransferase, WD-40 repeat proteins linked to immune signalling, defence regulation, or metabolism (Wang et al., 2024). Several QTL that correspond to or are likely identical to *Yr30* include *QYr.cim-3BS* (Chapio and Francolin#1) (Yang et al., 2013; Pretorius et al., 2016), *QYr.spa-3B.1/ QSr.spa-3B.1/ QPbc.spa-3B.1* (AC Cadillac and Red Fife) (Singh et al., 2013; Singh et al., 2014; Bokore et al., 2017), *QYr.inra-3BS* (Renan) (Dedryver et al., 2009), *Qyrlov.nwafu-3BS* (P10057) (Wu et al., 2017), *QYr.nwafu-3BS* (P9936) (Huang et al., 2019), *QYr.hbaas-3BS* (Jia et al., 2020), *QYr.lrdc-3B* (P2711) (Farzand et al., 2022) and *QYrr-3B* (Opata 85) (Chhuneja et al., 2006).

An ASR gene, *Yr57 (YrAW2)*, was identified in the common wheat landrace ‘AUS27858’ from the Watkins collection and was effective against several pathotypes, including Pst pathotype 134 E16A+Yr17+Yr27+. A test for allelism with Rubric (carrying *Yr4*) showed that the gene is distinct from *Yr4* (Randhawa et al., 2015). The ASR gene, *YrRub*, was identified in the Australian cultivar ‘Rubric’ derived from the CIMMYT line EMU ‘S’. Genotypes carrying *Yr4,* e.g. Hybrid 46 and Avalon, amplified the same alleles as barc75 and cfb3530, suggesting *YrRub* is likely *Yr4,* though tests for allelism were not conducted (Bansal et al., 2009). An ASR gene *Yr58 (YrW195) (QYr.sun-3BS)* was identified in the landrace ‘W195’, conferring resistance at the fourth leaf stage and was confirmed to be different from other genes using race specificity tests (Chhetri et al., 2016a; Chhetri et al., 2016b).

Other QTL identified in this cluster include an ASR QTL *QYr.nwafu-3BS* (Wu et al., 2020) and loci identified in field studies; *QYrhm.nwafu-3BS* (Humai 15) (Yuan et al., 2018), *QLr-3BS/QYr-3BS* (Zhang et al., 2021), *QLr.ifa-3BS/QYr.ifa-3BS* (Capo) (Buerstmayr et al., 2014), *QYrsk.wgp-3BS* (Skiles) (Liu et al., 2019b), *QYrto.swust-3BS* (Toni) (Zhou et al., 2019) and *QYr.cim-3BS* (Mucuy) (Liu et al., 2022a).

Candidate gene prediction within the region of *QYrto.swust-3BS* (Toni) revealed 14 genes encoding protein kinases (Zhou et al., 2019) while four genes encoding glycosyltransferases and one ATP-binding cassette gene were identified within the region of *QYr.hbaas-3BS* (Jia et al., 2020).

***Yr80***

The APR gene *Yr80 (YrAW11)* was identified in a landrace ‘Aus27284’ and was effective against Australian Pst isolates. It showed resistance to leaf rust and stem rust (Ug99 and derivatives), suggesting potential pleiotropy or tight linkage (Nsabiyera et al., 2018).

**C3B.2 (757.3 – 791.26 Mb)**

An ASR gene, *Yr82,* was identified in the Portuguese landrace ‘Aus27969’ from the Watkins collection against several Pst pathotypes and displayed low infection types in both seedling (IT 1–12C) and field tests in Australia. (Pakeerathan et al., 2019). QTL located in this cluster include an ASR locus *QYr.nwafu-3BL* (Wu et al., 2020) and two loci from field studies; *QYr-3BL* (Zhang et al., 2021) and *QYr.sun-3B* identified in the Australian durum cv. ‘Wollarroi’ (Bansal et al., 2013).

**C3B.3 (823.06 – 839.48 Mb)**

This cluster consists of loci detected in adult stages, including *QYrex.wgp-3BL* (Express) (Lin and Chen, 2008) and *QYr.nmbu.3B* identified in a GWAS study of a Nordic spring panel (Lin et al., 2023).

*QYr.rcrrc.3B.1* was identified in a DH population from the IWWIP advanced line ‘130675’, potentially linked to the ASR gene Yr4 at 5.58 Mb based on race specificity tests (Tehseen et al., 2022). However, projection of its closely linked SNP marker 5971264 on CS v2.1 placed it on 3BL at 838.13 Mb.

**Chromosome 4B**

**4B (1.32 – 15.03 Mb)**

This cluster includes QTL that confer resistance at the adult stage; *QYr.spa-4B* (Carberry and Lillian) (Bokore et al., 2017) and *QYr.hebau-4BS* (Fuyu3) (Gebrewahid et al., 2020a) and *QYr.hebau-4BS* (Thatcher) (Zhang et al., 2019b).

***Yr50***

The ASR gene, *Yr50,* was identified in a wheat introgression line ‘CH223’ derived from *Thinopyrum intermedium*-derived partial amphiploid (TAI7047) (Liu et al., 2013a). Genomic in situ hybridisation (GISH) revealed no detectable *T. intermedium* chromosomal segments in CH223, suggesting a cryptic or very small translocation (Liu et al., 2013a).

**C4B.2 (525.99 – 588.18 Mb)**

The APR gene *Yr62* was identified in the accession ‘PI 192252’ and mapped between the SSR markers Xgwm192 and Xgwm251 (Lu et al., 2014). Yr62 confers HTAP resistance, exhibiting strong resistance (IT 1–2) at adult-plant stages under elevated temperatures (10–30°C) (Lu et al., 2014). Two QTL share the flanking marker Xgwm251 with Yr62, namely *QYrhm.nwafu-4BL* from ‘Humai 15’ (Yuan et al., 2018) and the HTAP QTL *QYrsk.wgp-4BL* from ‘Skiles’ (Liu et al., 2019b), suggesting that this region may represent an important APR resistance hotspot.

The APR gene *Yr68*, identified in the accession ‘AGG91587WHEA1’, is also located within this cluster and is flanked by markers IWB74301 and IWB28394 (McIntosh, 2024). Additional loci mapped to this region include *QYr.caas-4BL* (Libellula) (Lu et al., 2009), *QYrus.vt-4BL* (USG 3555) (Christopher et al., 2012), *QYr.hbaas-4BL.1*, *QYr.hbaas-4BL.2* and *QYr.hbaas-4BL.3* identified through GWAS (Jia et al., 2020), QYr-4BL.1 and QYr-*4BL.2* (Zhang et al., 2021) and *QYr.inra-4B* (Apache) (Paillard et al., 2012).

**C4B.3 (608.37 – 666.89 Mb)**

The cluster contains *QYr.nwafu-4BL* (*YrRC*), identified through GWAS and reported to confer all-stage resistance (Wu et al., 2020). *QYr.crc-4BL* was identified in the cultivar ‘Thatcher’ in a field-based study, and candidate resistance genes encoding NB-LRR proteins and protein kinase domains (Rosa et al., 2019). Other loci within this interval include *QYr.caas-4BS* from ‘Dabaimai’ (Wang et al., 2021a), *QYr.nwafu-4BL.2* from ‘Fengdecun 12’ (Liu et al., 2022b), and *QYr.spa-4B* detected in both ‘Carberry’ and ‘Red Fife’ (Bokore et al., 2017). Additional loci include QYr.gaas.4B and QYr.gaas.4B.1 from ‘Guixie 3’ (Cheng et al., 2022) and QYrst.orr-4BL from ‘Stephens’ (Vazquez et al., 2012).

**Chromosome 5B**

**C5B.1 (8.25 – 55.28 Mb)**

An ASR gene, *Yr47* (YrW1), was identified in two Iranian landraces, ‘AUS28183’ and ‘AUS28187’, from the Watkins collection against the Australian Pst pathotypes 134 E16A+ and 134 E16A+Yr17+. Nulli-tetrasomic lines were used to confirm the 5B-specificity of Xgwm234. It was found to be tightly linked (3-6 cM) to the leaf rust resistance gene *Lr52*(*LrW1*) (Bansal et al., 2011). Using a high-resolution mapping population, Yr47 was mapped 0.2 cM from Lr52, and the SSR marker sun180 was mapped 0.4cM from *Lr52* (Qureshi et al., 2017). *QYr.inra-5BL.2* identified in the cv. ‘Camp Rémy’ is flanked by the markers Xgwm234 and XDuPw115a (Mallard et al., 2005). Camp Rémy likely carries the 5BS/7BS translocation inherited from the cv. Cappelle Desprez (Mallard et al., 2005). Xgwm234 is part of a major gene cluster that includes a putative nucleotide-binding site-leucine-rich repeat (NBS-LRR) gene (sequence BM136556a) (Sourdille et al., 2004; Mallard et al., 2005).

The QTL *QYr.nwafu-5BS* and *qNV.Yr-5B.1* identified in GWAS studies are likely the gene *Yr47* based on positional alignment (Wu et al., 2020; Jambuthenne et al., 2022).

Other loci in this cluster include the ASR QTL *QYrel.wgp-5BS* (Eltan) (Liu et al., 2019a) and field detected QTL *QYr.uga-5B* (Pioneer 26R61) (Hao et al., 2011), *qNV.Yr-5B.2* and *qNV.Yr-5B.3* (Jambuthenne et al., 2022).

***Yr74***

The seedling resistance specificity temporarily designated *YrA* was first reported in Australian and CIMMYT-derived spring wheats following the introduction of *Puccinia striiformis* f. sp. *tritici* into Australia in 1979 (Wellings et al., 1988). Early studies noted substantial genetic heterogeneity among resistant lines and a strong environmental influence on expression, with resistance requiring high light intensity post-inoculation and being suppressed under low-light conditions, indicating pronounced genotype × environment (G×E) interaction (Wellings et al., 1988). Subsequent genetic dissection using a Teal × Avocet R doubled haploid population demonstrated that the *YrA* phenotype is conditioned by two complementary dominant genes, *Yr73* located on chromosome arm 3DL and *Yr74* on 5BL (Dracatos et al., 2016). Importantly, neither locus alone confers resistance; the characteristic *YrA* seedling resistance response is expressed only when both genes are present, illustrating a rare example of complementary gene action underlying stripe rust resistance (Dracatos et al., 2016).

**C5B.2 (507.26 – 603.79 Mb)**

This cluster consists of QTL detected in the adult stage, including *QYr.inra-5BL.1* (Camp Remy), *QYr.caas-5BL.1* and *QYr.caas-5BL.2* (Libellula) (Lu et al., 2009), *QYr.nmbu.5B.1* (Lin et al., 2023), *QYr.YBZR-5BL* (YBZR) (Deng et al., 2022), *QYrns.orz-5BL* (NSA-98-0995) (Vazquez et al., 2015b), *QYr.spa-5B* (Stettler) (Bokore et al., 2017), *QYr.AYH-5BL* (Anyuehong) (Long et al., 2021), *QYr.dms-5B* (Attila) (Zou et al., 2017), *QYrPI181410.wgp-5BL.2* (PI 181410) (Liu et al., 2020b), and *QYr.nwafu-5BL.1* (Fengdecun 12) (Liu et al., 2022b).

Potential pleiotropic or closely linked loci include *QYr-5BL.1/ QLr-5BL.1* (Zhang et al., 2021) and *QYr.caas-5BL.3* (Shanghai 3/Catbird) with a QTL for Fusarium head blight (Ren et al., 2012b).

**C5B.3 (661.71 – 698.07 Mb)**

This contains an ASR QTL *QYrdr.wgp-5BL.1* (Druchamp) (Hou et al., 2015) and several adult stage detected QTL, including *QYr.sun-5B* (Wollaroi) (Bansal et al., 2013), *QYr.nwafu-5BL.2* (Fengdecun 12) (Liu et al., 2022b), *Qyr.gaas-5B* (Guixie 3) (Cheng et al., 2022) and *QYr-5BL.2* (Zhang et al., 2021).

Potential pleiotropy was observed for the QTL *QYr.spa-5B* (AC Cadillac), which co-localised with powdery mildew resistance (*QPM.spa-5B*) and leaf tip necrosis (*QLTN.spa-5B*) QTL (Bokore et al., 2017). In a different study, *QYr.spa-5B* also overlapped with an epistatic stem rust resistance locus (Singh et al., 2014; Bokore et al., 2017). The marker Xbarc59 is closely linked to *QYr.cim-5BL* (Kenya Kongoni) and a leaf rust resistance QTL, *QLr.fcu-5BL,* reported by Chu et al. (2009), suggesting a potential pleiotropic or closely linked locus (Calvo-Salazar et al., 2015).

Candidate gene prediction for the QTL *QYr.usw-5B* identified in the durum cv ‘W9262-260D3’ revealed two leucine-rich repeat receptor-like protein kinases within its physical interval (Lin et al., 2018).

**Chromosome 6B**

**C6B.1 (5.29 – 40.48 Mb)**

The ASR gene *Yr35* was transferred from *Triticum dicoccoides* accession 479 to common wheat line ‘98M71’ in a 'Chinese Spring' background and is closely linked to *Lr53* (Marais et al., 2005). The QTL *qNV.Yr-6B.1 and qNV.Yr-6B.2* identified in a GWAS study in Australia, co-locates with *Yr35* (Jambuthenne et al., 2022). The cluster also includes a pleiotropic locus *QYr.caas-6BS.3/QLr.caas-6BS.1*, identified in the cv. ‘Bainong 64’ (Ren et al., 2012c).

Other QTL in this cluster are an ASR *QYrdr.wgp-6BL.1* (Druchamp) (Hou et al., 2015) and field detected QTL *QYr-6BS* (Zhang et al., 2021), *QYrqin.nwafu-6BS* (Qinnong 142) (Zeng et al., 2019b) and *QYr.caas-6BS.1* (Ibis) (Bai et al., 2012).

**C6B.2 (65.71 – 149.39 Mb)**

The APR gene *Yr78 (QYr.ucw-6B)* was initially identified in a GWAS study of the USDA-ARS spring wheat collection (Maccaferri et al., 2015; Dang et al., 2022). It confers partial resistance, and it was validated in 10 populations from resistant accessions from the same collection (Dang et al., 2022). The QTL *QYrsn.nwafu-6BS* (Shaannong 33) (Huang et al., 2021), *QYr.cim-6BS* (Mucuy) (Liu et al., 2022a), *QYrst.wgp-6BS.2* (Stephens) (Santra et al., 2008) and *QYrCW357-6BS* (Changwu357-9) (Huang et al., 2022) are reported to be potentially *Yr78*. *QYrsk.wgp-6B* was reported to potentially be *Yr78*, though slight marker allele differences suggest possible allelic variation (Liu et al., 2019b). Although the QTL *QYr.nmbu.6B* occurs in proximity, its race-specific nature contrasts with the race non-specific resistance profile of *Yr78* (Lin et al., 2023).

The HTAP gene, *Yr36,* was identified in *T. turgidum* ssp. *dicoccoides* (DIC) accession ‘FA15-3’ conferring moderate, durable resistance to Pst under high temperatures (Uauy et al., 2005). *Yr36* is closely linked to the grain protein content locus *Gpc-B1* (Uauy et al., 2005; Fu et al., 2009). Map-based cloning of *Yr36* revealed that it encodes a WKS1 protein (Wheat Kinase START1), comprising a kinase domain and a lipid-binding START domain (Fu et al., 2009). The QTL *QYr.spa-6B* (Lilian) and *qNV.Yr-6B.3*, are reported to be likely *Yr36* based on marker proximity to *Gpc-B1* and HTAP profile, respectively (Bokore et al., 2017; Jambuthenne et al., 2022).

***Yr83***

*Yr83* is an ASR gene located on rye chromosome 6R, which was introgressed into wheat from triticale line ‘T-701’. Seedling tests in Australia with prevalent Pst pathotypes showed T-701 and a wheat-rye 6R(6D) disomic substitution line (Sub6R(6D)) as highly resistant (IT 0; to 1C) (Li et al., 2020; Li et al., 2022).

**Chromosome 7B**

***Yr63***

The ASR gene, *Yr63*, from the Portuguese landrace ‘AUS27955’ (Watkins Collection) was shown to confer resistance against multiple pathotypes from Australia, Africa, Asia, Europe, the Middle East, and South America, except for the Australian pathotype *Pst* S10 (239 E237 A- 17+ 33+) (Mackenzie et al., 2023). It is positioned in a hot spot enriched with multiple nucleotide-binding (NB) and leucine-rich repeat (LRR) and kinase domain encoding genes (Mackenzie et al., 2023).

***Yr79***

The gene *Yr79* was identified in a Pakistani landrace ‘PI 182103’ and expressed under high temperatures (Feng et al., 2018).

**C7B.1 (625.79 – 657.16 Mb)**

*Yr39* was identified in the cv ‘Alpowa’ conferring non-race-specific HTAP resistance in the U.S. (Lin and Chen, 2007). *QYr-7BL.1* (Zhang et al., 2021) and *QYr.spa-7B.1* (AC Cadillac) (Singh et al., 2014) have been reported as being closely linked or allelic variants of *Yr39*. The pleiotropic locus *QYr.cim-7BL/QLr.cim-7BL* (Kenya Kongoni) may be associated with *Yr39*, as marker wPt-9925 was previously linked to *Yr39*, though Xgwm131 (closely linked to *Yr39*) was monomorphic in this population (Calvo-Salazar et al., 2015).

**C7B.2 (686.15 – 755.05 Mb)**

The ASR gene, *Yr6*, was identified in the 1960’s by Macer (1966) and is found in many cultivars globally (McIntosh et al., 1995). A study by Ma et al. (2015) identified *Yr6* in a UK cv. ‘Cadenza’ as closely linked to Xgwm577. Recently, *Yr6* has been cloned in two different studies using the cv. ‘Aikang 58 (AK58)’ and ‘CDC_Stanley’ (Huang et al., 2025; Wu et al., 2025). In both cases, it was reported to encode a dual-function NLR (sensor and executor NLRs) in a head-to-head orientation. *Yr6* in AK58 was found to be allelic to a powdery mildew resistance locus *Pm5* originally identified in the cv. ‘Fuzhuang 30’ (Guo et al., 2025; Sun et al., 2025; Wu et al., 2025). The ASR QTL *QYr.caas-7BL.1* identified in the moderately susceptible cross ‘Shanghai 3/Catbird’ is also flanked by the marker Xgwm577 (Ren et al., 2012b).

The ASR gene *Yr67* (*YrVL2*) was identified in the Indian cv. ‘VL Gehun 892 (VL892)’ in Australia and was effective against predominant Pst pathotypes (Bariana et al., 2022). It is likely identical to *YrC591* identified in the cv. ‘C591’ (present in VL892’s pedigree) (Li et al., 2009; Bariana et al., 2022).

Other ASR QTL in this cluster include *QYr.nwafu-7BL* (Wu et al., 2020), *QYr.sicau-7BL* (Shumai126) (Wang et al., 2022), *QYr.usw-7B* (W9262-260D3) (Lin et al., 2018).

The APR gene *Yr59* was identified in the cv. ‘PI 178759’ from Iraq and expressed under high temperatures. The gene also showed partial resistance in high-temperature seedling tests (Zhou et al., 2014b). The APR gene *Yr52* was identified in an Indian cv. ‘PI 183527’ and expressed under high temperatures (Ren et al., 2012a). The HTAP QTL *qNV.Yr-7B.4* was reported to be either *Yr52* or *Yr59* based on its detection under high temperatures (Jambuthenne et al., 2022).

This cluster also includes potential pleiotropic loci; *QYr.hebau-7BL/ QLr.hebau-7BL(Lr68)* (Fuyu3) (Gebrewahid et al., 2020a), *QYr.hebau-7BL/QLr.hebau-7BL(Lr68)* (SW 8588) (Zhang et al., 2019b), *QYr.hbau-7BL/QLr.hbau-7BL(Lr68)* (Mianyang351-15) (Gebrewahid et al., 2020b) and *QLr.cim-7BL/QYr.cim-7BL* (KU3067) (Zhang et al., 2022).

Other loci conferring resistance at the adult stage in this cluster include *QYr-7BL.2* (Zhang et al., 2021), *QYr.nwafu-7BL* (P9936) (Huang et al., 2019), *QYr.spa-7B.2* (AC Cadillac) (Singh et al., 2014), *QY.hbaas-7BL* (Jia et al., 2020), *QYrcen.nwafu-7BL* (Centrum) (Mu et al., 2019) and *QYr.hebau-7BL* (Fundulea 900) (Yan et al., 2021).

***Yr/QYr* Clusters on D Sub-Genome**

**Chromosome 1D**

**C1D.1 (0.44 – 16.98 Mb)**

Two ASR loci occur in this cluster, *QYr.nwafu-1DS.1,* identified in a GWAS study (Wu et al., 2020) and *QYrsn.nwafu-1DS* identified in ‘Shaannong 33’ (Huang et al., 2021). Several QTL conferring resistance in the adult stage occur in this cluster. They include *QYryz.caas-1DS* (Zhongmai 895) (Zhu et al., 2023), *QYrdr.wgp-1DS* (Druchamp) (Hou et al., 2015), *QYrst.orr-1DS* (Stephens) (Vazquez et al., 2012), *Qyr.gaas.1D.1* and *Qyr.gaas.1D* (Guixie 3) (Cheng et al., 2022) and *Qyr.nwafu-1DS* (Jimai 229) (Liu et al., 2023). *QYr-1DS* (Zhang et al., 2021) and *QYr.hbaas-1DS* (Jia et al., 2020) were identified from GWAS studies, with gene prediction revealing that *QYr.hbaas-1DS* may encode RPM1 and NBS-LRR protein candidates (Jia et al., 2020).

The only formally named gene on chromosome 1D is the ASR gene, *Yr25*, identified in a French wheat line ‘TP1295’ using monosomic analysis and was effective against the Pst isolate WYR 85-22 (race 6E0) (Calonnec and Johnson, 1998; Calonnec et al., 2002). *Yr25* has been shown to be present in cv. Heines VII, Strubes Dickkopf, Carstens V and Spaldings Prolific used in differential sets for Pst seedling gene postulation (Calonnec et al., 2002). Due to a lack of marker information for Yr25, it was not possible to determine its physical location on the reference genome. Thus, fine mapping and allelism tests are needed to resolve relationships within this cluster.

**Chromosome 2D**

**C2D.1 (5.92 – 36.21 Mb)**

This cluster includes an ASR locus *QYr.nwafu-2DS* (Wu et al., 2020)*,* and two loci *QYr.caas-2DS* (Libellula) (Lu et al., 2009) and *QYr.nmbu.2D* (Lin et al., 2023) that confer resistance at the adult stage. A potentially pleiotropic locus *QYr.hbau-2DS*/*QLr.hbau-2DS* (Mianyang351-15) (Gebrewahid et al., 2020b) also occurs in this cluster.

**C2D.2 (67.0 – 90.18 Mb)**

The APR gene *Yr16* was originally identified in the cv. ‘Cappelle-Desprez’ and its location confirmed by cytogenetic studies (Worland, 1986; Worland et al., 1988). The QTL *QYr.ufs-2D*, was identified in the cv. ‘Yr16DH70 (a Cappelle-Desprez-derived line lacking seedling resistance genes *Yr3a* and *Yr4a*)’ flanked by markers Xgwm102 and wPt-664520 likely corresponds to the *Yr16* (Agenbag et al., 2012). *QYr*.*inra*-*2DS,* identified the cv. ‘Camp Rémy (CR), a Cappelle-Desprez descendant, is also flanked by the marker Xgwm102 (Mallard et al., 2005). An APR QTL, *QYr.caas-2DS.2*, identified in the Chinese cultivar ‘Lumai 21’, was mapped to the same 2DS resistance region and was considered by Ren et al. (2015) to be closely associated with the *Yr16* region, although its effect was smaller than that reported for *QYr.inra-2DS*.

***Yr54***

*Yr54* (*QYr.tam-2D*) is an APR gene identified in a CIMMYT wheat line ‘Quaiu 3’ in Mexico, explaining up to 54% phenotypic variation (Basnet et al., 2013).

**Chromosome 3D**

***Yr66***

*Yr66* is a seedling resistance gene identified in an Indian cv. ‘VL Gehun 892’ against Pst pathotype 134 E16A+Yr17+Yr27+ (Bariana et al., 2022).

***Yr49***

*Yr49* is a classic example of a race-specific APR. It was originally identified in the cv. ‘AUS91433’ as an APR gene providing resistance to Australian Pst pathotypes, but it succumbed to Chinese Pst pathotypes (Ellis et al., 2014; McIntosh, 2024).

***Yr45***

*Yr45* is an ASR gene identified in an Afghan cv. ‘PI 181434’ and is highly effective against all N. American Pst pathotypes (Li et al., 2011).

**C3D.1 (62.48 – 80.2 Mb)**

This cluster includes a potentially pleiotropic locus *QYr.cim-3DC/QLr.cim-3DC* (UC1110) (Lan et al., 2017) and two loci *QYr.ucw-3D* (Klein Chajá) (Cobo et al., 2018) and *QYr.cim-3DS* (Mucuy) (Liu et al., 2022a) that confer resistance at the adult stage.

***Yr71***

The APR gene *Yr71 (YrSA3)* was identified in the Australian cv. ‘Sunco’ is tightly linked to leaf rust and stem rust resistance complex *Lr24/Sr24* (Bariana et al., 2016).

**Chromosome 4D**

***Yr28***

The ASR gene *Yr28* was identified in a CIMMYT synthetic cv. ‘Opata 85’ against Mexican pathotypes 14E14 and MEX96-11. The positive allele was the *Ae. tauschii* parent (Singh et al., 2000).

Map-based cloning of the gene *YrAS2388 (Yr28)* identified in *Ae. tauschii* cv. ‘PI511383, CIae9 and PI511384’ revealed that it encodes an NB-LRR gene with duplicated 3’ untranslated regions (3’UTRs) (Liu et al., 2013b; Zhang et al., 2019a).

***Yr46***

The APR gene *Yr46* was initially identified in the cv ‘Pavon76’ and is pleiotropic to *Lr67/Sr55/Pm46/Ltn3* (Herrera-Foessel et al., 2011; Herrera-Foessel et al., 2014; Moore et al., 2015). Map-based cloning of this locus revealed that it encodes a sugar (hexose) transporter rather than an immune receptor (Moore et al., 2015).

**C4D.1 (439.53 – 478.05 Mb)**

This cluster includes loci that may be linked to the pleiotropic locus Yr46, conferring resistance at the adult stage, and includes *QYr.nmbu.4D* (Lin et al., 2023) and *QYr.caas-4DL* (Bainong 64) (Ren et al., 2012c). *QYr.caas-4DL* co-located with a powdery mildew QTL *QPm.caas-4DL,* but leaf rust was not detected (Ren et al., 2012c).

The other QTL in this cluster include *qNV.Yr-4D.2* (Jambuthenne et al., 2022) and *QYr.hbaas-4DL* (Jia et al., 2020) identified in GWAS studies. Candidate gene prediction within the region of *QYr.hbaas-4DL* revealed that it may potentially encode receptor-like kinase and kinase genes (Jia et al., 2020).

**Chromosome 5D**

***Yr70***

The seedling resistance gene *Yr70 (YrUmb)* was identified in an *A. umbellulata* introgression line ‘IL 393-4’, and it co-segregates with the leaf rust resistance gene *Lr76 (LrUmb)* (Bansal et al., 2016). Candidate gene prediction revealed that it may potentially encode NB-LRR genes (Bansal et al., 2020).

**Chromosome 6D**

***Yr77***

The APR gene *Yr77 (QYr.ucw-6D)* was identified in a GWAS of a global collection in the US against diverse Pst races and was subsequently confirmed in the cv. ‘PI 322118, PI 164377, PI 388095, PI 520350 and PI 623378’ (Maccaferri et al., 2015).

**Chromosome 7D**

**C7D.1 (48.95 – 76.44 Mb)**

The APR gene *Yr18* was originally identified *Lr34*-Thatcher NILs and it is pleiotropic to *Lr34/Sr57/Pm38/Ltn1* (Singh, 1992; Ma and Singh, 1996; Suenaga et al., 2003; Wu et al., 2015). Map-based cloning of this locus revealed that it encodes an ATP-binding cassette (ABC) transporter of the pleiotropic drug resistance sub-family (Krattinger et al., 2009).

All the QTL in this cluster are reported to be identical to the *Yr18* complex and include *QYr.YBZR-7DS* (Yibinzhuermai (YBZR)) (Deng et al., 2022), *QYr.hebau-7DS* (Fundulea 900) (Yan et al., 2021), *QYr.caas-7DS* (Libellula and Strampelli) (Lu et al., 2009), *QYr.spa-7D* (Lillian and Carberry) (Bokore et al., 2017), *QYr.AYH-7DS* (Anyuehong (AYH)) (Long et al., 2021), *QYr.sgi-7D* (Kariega) (Ramburan et al., 2004), *QYr.GX-7DS* (Gaoxianguangtoumai (GX)) (Wang et al., 2021b), *qNV.Yr-7D.1* (Jambuthenne et al., 2022), and *QYrmx.nwafu-7DS* (Mingxian 169) (Yuan et al., 2018).

**References**

Agenbag, G.M., Pretorius, Z.A., Boyd, L.A., Bender, C.M., MacCormack, R., and Prins, R. (2014). High-resolution mapping and new marker development for adult plant stripe rust resistance QTL in the wheat cultivar Kariega. *Molecular Breeding* 34(4)**,** 2005-2020. doi: 10.1007/s11032-014-0158-4.

Agenbag, G.M., Pretorius, Z.A., Boyd, L.A., Bender, C.M., and Prins, R. (2012). Identification of adult plant resistance to stripe rust in the wheat cultivar Cappelle-Desprez. *Theor Appl Genet* 125(1)**,** 109-120. doi: 10.1007/s00122-012-1819-5.

Athiyannan, N., Abrouk, M., Boshoff, W.H.P., Cauet, S., Rodde, N., Kudrna, D., et al. (2022). Long-read genome sequencing of bread wheat facilitates disease resistance gene cloning. *Nat Genet* 54(3)**,** 227-231. doi: 10.1038/s41588-022-01022-1.

Bai, B., Ren, Y., Xia, X.-c., Du, J.-y., Zhou, G., Wu, L., et al. (2012). Mapping of Quantitative Trait Loci for Adult Plant Resistance to Stripe Rust in German Wheat Cultivar Ibis. *Journal of Integrative Agriculture* 11(4)**,** 528-536. doi: 10.1016/s2095-3119(12)60039-2.

Bansal, M., Adamski, N.M., Toor, P.I., Kaur, S., Molnar, I., Holusova, K., et al. (2020). *Aegilops umbellulata* introgression carrying leaf rust and stripe rust resistance genes *Lr76* and *Yr70* located to 9.47-Mb region on 5DS telomeric end through a combination of chromosome sorting and sequencing. *Theor Appl Genet* 133(3)**,** 903-915. doi: 10.1007/s00122-019-03514-x.

Bansal, M., Kaur, S., Dhaliwal, H.S., Bains, N.S., Bariana, H.S., Chhuneja, P., et al. (2016). Mapping of *Aegilops umbellulata*‐derived leaf rust and stripe rust resistance loci in wheat. *Plant Pathology* 66(1)**,** 38-44. doi: 10.1111/ppa.12549.

Bansal, U.K., Forrest, K.L., Hayden, M.J., Miah, H., Singh, D., and Bariana, H.S. (2011). Characterisation of a new stripe rust resistance gene *Yr47* and its genetic association with the leaf rust resistance gene *Lr52*. *Theor Appl Genet* 122(8)**,** 1461-1466. doi: 10.1007/s00122-011-1545-4.

Bansal, U.K., Hayden, M.J., Gill, M.B., and Bariana, H.S. (2009). Chromosomal location of an uncharacterised stripe rust resistance gene in wheat. *Euphytica* 171(1)**,** 121-127. doi: 10.1007/s10681-009-0007-4.

Bansal, U.K., Kazi, A.G., Singh, B., Hare, R.A., and Bariana, H.S. (2013). Mapping of durable stripe rust resistance in a durum wheat cultivar Wollaroi. *Molecular Breeding* 33(1)**,** 51-59. doi: 10.1007/s11032-013-9933-x.

Bariana, H., Forrest, K., Qureshi, N., Miah, H., Hayden, M., and Bansal, U. (2016). Adult plant stripe rust resistance gene *Yr71* maps close to *Lr24* in chromosome 3D of common wheat. *Molecular Breeding* 36(7). doi: 10.1007/s11032-016-0528-1.

Bariana, H., Kant, L., Qureshi, N., Forrest, K., Miah, H., and Bansal, U. (2022). Identification and Characterisation of Stripe Rust Resistance Genes *Yr66* and *Yr67* in Wheat Cultivar VL Gehun 892. *Agronomy* 12(2). doi: 10.3390/agronomy12020318.

Bariana, H.S., and McIntosh, R.A. (1993). Cytogenetic studies in wheat. XV. Location of rust resistance genes in VPM1 and their genetic linkage with other disease resistance genes in chromosome 2A. *Genome* 36(3)**,** 476-482. doi: 10.1139/g93-065.

Bariana, H.S., Parry, N., Barclay, I.R., Loughman, R., McLean, R.J., Shankar, M., et al. (2006). Identification and characterization of stripe rust resistance gene *Yr34* in common wheat. *Theor Appl Genet* 112(6)**,** 1143-1148. doi: 10.1007/s00122-006-0216-3.

Basnet, B.R., Singh, R.P., Ibrahim, A.M.H., Herrera-Foessel, S.A., Huerta-Espino, J., Lan, C., et al. (2013). Characterization of *Yr54* and other genes associated with adult plant resistance to yellow rust and leaf rust in common wheat Quaiu 3. *Molecular Breeding* 33(2)**,** 385-399. doi: 10.1007/s11032-013-9957-2.

Bobrowska, R., Noweiska, A., Spychala, J., Tomkowiak, A., Nawracala, J., and Kwiatek, M.T. (2022). Diagnostic accuracy of genetic markers for identification of the *Lr46/Yr29* "slow rusting" locus in wheat (*Triticum aestivum* L.). *Biomol Concepts* 13(1)**,** 1-9. doi: 10.1515/bmc-2022-0002.

Bokore, F.E., Cuthbert, R.D., Knox, R.E., Randhawa, H.S., Hiebert, C.W., DePauw, R.M., et al. (2017). Quantitative trait loci for resistance to stripe rust of wheat revealed using global field nurseries and opportunities for stacking resistance genes. *Theor Appl Genet* 130(12)**,** 2617-2635. doi: 10.1007/s00122-017-2980-7.

Buerstmayr, M., Matiasch, L., Mascher, F., Vida, G., Ittu, M., Robert, O., et al. (2014). Mapping of quantitative adult plant field resistance to leaf rust and stripe rust in two European winter wheat populations reveals co-location of three QTL conferring resistance to both rust pathogens. *Theor Appl Genet* 127(9)**,** 2011-2028. doi: 10.1007/s00122-014-2357-0.

Calonnec, A., and Johnson, R. (1998). Chromosomal Location of Genes for Resistance to *Puccinia striiformis* in the Wheat Line TP1295 Selected from the Cross of Soissonais-Desprez with Lemhi. *European Journal of Plant Pathology* 104(8)**,** 835-847. doi: 10.1023/a:1008660904975.

Calonnec, A., Johnson, R., and De Vallavieille‐Pope, C. (2002). Genetic analyses of resistance of the wheat differential cultivars Carstens V and Spaldings Prolific to two races of *Puccinia striiformis*. *Plant Pathology* 51(6)**,** 777-786. doi: 10.1046/j.1365-3059.2002.00788.x.

Calvo-Salazar, V., Singh, R.P., Huerta-Espino, J., Cruz-Izquierdo, S., Lobato-Ortiz, R., Sandoval-Islas, S., et al. (2015). Genetic Analysis of Resistance to Leaf Rust and Yellow Rust in Spring Wheat Cultivar Kenya Kongoni. *Plant Dis* 99(8)**,** 1153-1160. doi: 10.1094/PDIS-07-14-0718-RE.

Cao, Q., Zhu, Z., Xu, D., Wu, J., Xu, X., Dong, Y., et al. (2024). Characterization of a 4.1 Mb inversion harboring the stripe rust resistance gene *Yr86* on wheat chromosome 2AL. *The Crop Journal* 12(4)**,** 1168-1175. doi: 10.1016/j.cj.2024.05.011.

Cao, X., Zhou, J., Gong, X., Zhao, G., Jia, J., and Qi, X. (2012). Identification and validation of a major quantitative trait locus for slow-rusting resistance to stripe rust in wheat. *J Integr Plant Biol* 54(5)**,** 330-344. doi: 10.1111/j.1744-7909.2012.01111.x.

Carter, A.H., Chen, X.M., Garland-Campbell, K., and Kidwell, K.K. (2009). Identifying QTL for high-temperature adult-plant resistance to stripe rust (*Puccinia striiformis* f. sp. *tritici*) in the spring wheat (*Triticum aestivum* L.) cultivar 'Louise'. *Theor Appl Genet* 119(6)**,** 1119-1128. doi: 10.1007/s00122-009-1114-2.

Chen, J., Chu, C., Souza, E.J., Guttieri, M.J., Chen, X., Xu, S., et al. (2011). Genome-wide identification of QTL conferring high-temperature adult-plant (HTAP) resistance to stripe rust (*Puccinia striiformis* f. sp. *tritici*) in wheat. *Molecular Breeding* 29(3)**,** 791-800. doi: 10.1007/s11032-011-9590-x.

Chen, S., Hegarty, J., Shen, T., Hua, L., Li, H., Luo, J., et al. (2021). Stripe rust resistance gene *Yr34* (synonym *Yr48*) is located within a distal translocation of *Triticum monococcum* chromosome 5A(m)L into common wheat. *Theor Appl Genet* 134(7)**,** 2197-2211. doi: 10.1007/s00122-021-03816-z.

Chen, X. (1995). Chromosomal Location of Genes for Resistance to *Puccinia striiformis* in Winter Wheat Cultivars Heines VII, Clement, Moro, Tyee, Tres, and Daws. *Phytopathology* 85(11). doi: 10.1094/Phyto-85-1362.

Chen, X., and Line, R.F. (1992). Genes for Resistance to Stripe Rust in ‘Tres’ Wheat. *Crop Science* 32(3)**,** 692-696. doi: 10.2135/cropsci1992.0011183X003200030024x.

Chen, X., Soria, M.A., Yan, G., Sun, J., and Dubcovsky, J. (2003). Development of Sequence Tagged Site and Cleaved Amplified Polymorphic Sequence Markers for Wheat Stripe Rust Resistance Gene *Yr5*. *Crop Science* 43(6)**,** 2058-2064. doi: 10.2135/cropsci2003.2058.

Cheng, B., Gao, X., Cao, N., Ding, Y., Chen, T., Zhou, Q., et al. (2022). QTL mapping for adult plant resistance to wheat stripe rust in M96-5 x Guixie 3 wheat population. *J Appl Genet* 63(2)**,** 265-279. doi: 10.1007/s13353-022-00686-z.

Cheng, P., and Chen, X.M. (2010). Molecular mapping of a gene for stripe rust resistance in spring wheat cultivar IDO377s. *Theor Appl Genet* 121(1)**,** 195-204. doi: 10.1007/s00122-010-1302-0.

Cheng, P., Xu, L.S., Wang, M.N., See, D.R., and Chen, X.M. (2014). Molecular mapping of genes *Yr64* and *Yr65* for stripe rust resistance in hexaploid derivatives of durum wheat accessions PI 331260 and PI 480016. *Theor Appl Genet* 127(10)**,** 2267-2277. doi: 10.1007/s00122-014-2378-8.

Chhetri, M., Bansal, U., Toor, A., Lagudah, E., and Bariana, H. (2016a). Genomic regions conferring resistance to rust diseases of wheat in a W195/BTSS mapping population. *Euphytica* 209(3)**,** 637-649. doi: 10.1007/s10681-016-1640-3.

Chhetri, M., Bariana, H., Kandiah, P., and Bansal, U. (2016b). *Yr58*: A New Stripe Rust Resistance Gene and Its Interaction with *Yr46* for Enhanced Resistance. *Phytopathology* 106(12)**,** 1530-1534. doi: 10.1094/PHYTO-04-16-0182-R.

Chhetri, M., Miah, H., Wong, D., Hayden, M., Bansal, U., and Bariana, H. (2023). Mapping of a Stripe Rust Resistance Gene *Yr72* in the Common Wheat Landraces AUS27506 and AUS27894 from the Watkins Collection. *Genes (Basel)* 14(11). doi: 10.3390/genes14111993.

Chhuneja, P., Kaur, S., Goel, R.K., and Dhaliwal, H.S. (2006). Mapping of leaf rust and stripe rust resistance QTLs in bread wheat x synthetic RIL population under Indian field

conditions. *Indian J. Crop Science* 1 (1-2)**,** 49-54.

Christopher, M.D., Liu, S., Hall, M.D., Marshall, D.S., Fountain, M.O., Johnson, J.W., et al. (2013). Identification and Mapping of Adult Plant Stripe Rust Resistance in Soft Red Winter Wheat VA00W‐38. *Crop Science* 53(3)**,** 871-879. doi: 10.2135/cropsci2012.02.0086.

Christopher, M.D., Liu, S., Hall, M.D., Marshall, D.S., Fountain, M.O., Johnson, J.W., et al. (2012). Identification and mapping of adult‐plant stripe rust resistance in soft red winter wheat cultivar ‘USG 3555’. *Plant Breeding* 132(1)**,** 53-60. doi: 10.1111/pbr.12015.

Chu, C.G., Friesen, T.L., Xu, S.S., Faris, J.D., and Kolmer, J.A. (2009). Identification of novel QTLs for seedling and adult plant leaf rust resistance in a wheat doubled haploid population. *Theor Appl Genet* 119(2)**,** 263-269. doi: 10.1007/s00122-009-1035-0.

Cobo, N., Pflüger, L., Chen, X., and Dubcovsky, J. (2018). Mapping QTL for Resistance to New Virulent Races of Wheat Stripe Rust from Two Argentinean Wheat Cultivars. *Crop Science* 58(6)**,** 2470-2483. doi: 10.2135/cropsci2018.04.0286.

Dang, C., Zhang, J., and Dubcovsky, J. (2022). High-resolution mapping of *Yr78*, an adult plant resistance gene to wheat stripe rust. *Plant Genome* 15(2)**,** e20212. doi: 10.1002/tpg2.20212.

de Vallavieille-Pope, C., Picard-Formery, H., Radulovic, S., and Johnson, R. (1990). Specific resistance factors to yellow rust in seedlings of some French wheat varieties and races of *Puccinia striiformis* Westend in France. *Agronomie* 10(2)**,** 103-113. doi: 10.1051/agro:19900202.

Dedryver, F., Paillard, S., Mallard, S., Robert, O., Trottet, M., Negre, S., et al. (2009). Characterization of genetic components involved in durable resistance to stripe rust in the bread wheat 'Renan'. *Phytopathology* 99(8)**,** 968-973. doi: 10.1094/PHYTO-99-8-0968.

Deng, M., Long, L., Cheng, Y., Yao, F., Guan, F., Wang, Y., et al. (2022). Mapping a stable adult-plant stripe rust resistance QTL on chromosome 6AL in Chinese wheat landrace Yibinzhuermai. *The Crop Journal* 10(4)**,** 1111-1119. doi: 10.1016/j.cj.2021.10.011.

Dibley, K., Jost, M., McIntosh, R., Lagudah, E., and Zhang, P. (2024). The wheat stripe rust resistance gene *YrNAM* is *Yr10*. *Nat Commun* 15(1)**,** 3291. doi: 10.1038/s41467-024-47513-z.

Dracatos, P.M., Zhang, P., Park, R.F., McIntosh, R.A., and Wellings, C.R. (2016). Complementary resistance genes in wheat selection 'Avocet R' confer resistance to stripe rust. *Theor Appl Genet* 129(1)**,** 65-76. doi: 10.1007/s00122-015-2609-7.

Ellis, J.G., Lagudah, E.S., Spielmeyer, W., and Dodds, P.N. (2014). The past, present and future of breeding rust resistant wheat. *Front Plant Sci* 5**,** 641. doi: 10.3389/fpls.2014.00641.

Eriksen, L., Afshari, F., Christiansen, M.J., McIntosh, R.A., Jahoor, A., and Wellings, C.R. (2004). *Yr32* for resistance to stripe (yellow) rust present in the wheat cultivar Carstens V. *Theor Appl Genet* 108(3)**,** 576. doi: 10.1007/s00122-003-1573-9.

Farzand, M., Dhariwal, R., Hiebert, C.W., Spaner, D., and Randhawa, H.S. (2022). QTL mapping for adult plant field resistance to stripe rust in the AAC Cameron/P2711 spring wheat population. *Crop Science* 62(3)**,** 1088-1106. doi: 10.1002/csc2.20741.

Feng, J., Wang, M., See, D.R., Chao, S., Zheng, Y., and Chen, X. (2018). Characterization of Novel Gene *Yr79* and Four Additional Quantitative Trait Loci for All-Stage and High-Temperature Adult-Plant Resistance to Stripe Rust in Spring Wheat PI 182103. *Phytopathology* 108(6)**,** 737-747. doi: 10.1094/PHYTO-11-17-0375-R.

Feng, J., Yao, F., Wang, M., See, D.R., and Chen, X. (2023). Molecular Mapping of *Yr85* and Comparison with Other Genes for Resistance to Stripe Rust on Wheat Chromosome 1B. *Plant Dis* 107(11)**,** 3585-3591. doi: 10.1094/PDIS-11-22-2600-RE.

Fu, D., Uauy, C., Distelfeld, A., Blechl, A., Epstein, L., Chen, X., et al. (2009). A kinase-START gene confers temperature-dependent resistance to wheat stripe rust. *Science* 323(5919)**,** 1357-1360. doi: 10.1126/science.1166289.

Gebrewahid, T.W., Zhang, P., Zhou, Y., Yan, X., Xia, X., He, Z., et al. (2020a). QTL mapping of adult plant resistance to stripe rust and leaf rust in a Fuyu 3/Zhengzhou 5389 wheat population. *The Crop Journal* 8(4)**,** 655-665. doi: 10.1016/j.cj.2019.09.013.

Gebrewahid, T.W., Zhou, Y., Zhang, P., Ren, Y., Gao, P., Xia, X., et al. (2020b). Mapping of Stripe Rust and Leaf Rust Resistance Quantitative Trait Loci in the Chinese Spring Wheat Line Mianyang351-15. *Phytopathology* 110(5)**,** 1074-1081. doi: 10.1094/PHYTO-08-19-0316-R.

Gerechter-Amitai, Z.K., van Silfhout, C.H., Grama, A., and Kleitman, F. (1989). *Yr15* — a new gene for resistance to Puccinia striiformis in *Triticum dicoccoides* sel. G-25. *Euphytica* 43(1-2)**,** 187-190. doi: 10.1007/bf00037912.

Gerechter-Amital, Z.K., and Grama, A. (1974). Inheritance of resistance to stripe rust (*Puccinia striformis*) in crosses between wild emmer (*Triticum dicoccoides*) and cultivated tetraploid and hexaploid wheats. I. *Triticum durum*. *Euphytica* 23(2)**,** 387-392. doi: 10.1007/bf00035883.

Gessese, M., Bariana, H., Wong, D., Hayden, M., and Bansal, U. (2019). Molecular Mapping of Stripe Rust Resistance Gene *Yr81* in a Common Wheat Landrace Aus27430. *Plant Dis* 103(6)**,** 1166-1171. doi: 10.1094/PDIS-06-18-1055-RE.

Guo, G., Bai, K., Hou, Y., Gong, Z., Zhang, H., Wu, Q., et al. (2025). The wheat NLR pair RXL/Pm5e confers resistance to powdery mildew. *Plant Biotechnol J* 23(4)**,** 1260-1276. doi: 10.1111/pbi.14584.

Guo, Q., Zhang, Z.J., Xu, Y.B., Li, G.H., Feng, J., and Zhou, Y. (2008). Quantitative trait loci for high-temperature adult-plant and slow-rusting resistance to *Puccinia striiformis* f. sp. *tritici* in wheat cultivars. *Phytopathology* 98(7)**,** 803-809. doi: 10.1094/PHYTO-98-7-0803.

Han, D.J., Wang, Q.L., Chen, X.M., Zeng, Q.D., Wu, J.H., Xue, W.B., et al. (2015). Emerging *Yr26*-Virulent Races of Puccinia striiformis f. tritici Are Threatening Wheat Production in the Sichuan Basin, China. *Plant Dis* 99(6)**,** 754-760. doi: 10.1094/PDIS-08-14-0865-RE.

Hao, Y., Chen, Z., Wang, Y., Bland, D., Buck, J., Brown-Guedira, G., et al. (2011). Characterization of a major QTL for adult plant resistance to stripe rust in US soft red winter wheat. *Theor Appl Genet* 123(8)**,** 1401-1411. doi: 10.1007/s00122-011-1675-8.

Herrera-Foessel, S.A., Lagudah, E.S., Huerta-Espino, J., Hayden, M.J., Bariana, H.S., Singh, D., et al. (2011). New slow-rusting leaf rust and stripe rust resistance genes *Lr67* and *Yr46* in wheat are pleiotropic or closely linked. *Theor Appl Genet* 122(1)**,** 239-249. doi: 10.1007/s00122-010-1439-x.

Herrera-Foessel, S.A., Singh, R.P., Lan, C.X., Huerta-Espino, J., Calvo-Salazar, V., Bansal, U.K., et al. (2015). *Yr60*, a Gene Conferring Moderate Resistance to Stripe Rust in Wheat. *Plant Dis* 99(4)**,** 508-511. doi: 10.1094/PDIS-08-14-0796-RE.

Herrera-Foessel, S.A., Singh, R.P., Lillemo, M., Huerta-Espino, J., Bhavani, S., Singh, S., et al. (2014). *Lr67/Yr46* confers adult plant resistance to stem rust and powdery mildew in wheat. *Theor Appl Genet* 127(4)**,** 781-789. doi: 10.1007/s00122-013-2256-9.

Hou, L., Chen, X., Wang, M., See, D.R., Chao, S., Bulli, P., et al. (2015). Mapping a Large Number of QTL for Durable Resistance to Stripe Rust in Winter Wheat Druchamp Using SSR and SNP Markers. *PLoS One* 10(5)**,** e0126794. doi: 10.1371/journal.pone.0126794.

Hou, L., Jia, J., Zhang, X., Li, X., Yang, Z., Ma, J., et al. (2016). Molecular Mapping of the Stripe Rust Resistance Gene *Yr69* on Wheat Chromosome 2AS. *Plant Dis* 100(8)**,** 1717-1724. doi: 10.1094/PDIS-05-15-0555-RE.

Huang, S., Liu, S., Zhang, Y., Xie, Y., Wang, X., Jiao, H., et al. (2021). Genome-Wide Wheat 55K SNP-Based Mapping of Stripe Rust Resistance Loci in Wheat Cultivar Shaannong 33 and Their Alleles Frequencies in Current Chinese Wheat Cultivars and Breeding Lines. *Plant Dis* 105(4)**,** 1048-1056. doi: 10.1094/PDIS-07-20-1516-RE.

Huang, S., Wu, J., Wang, X., Mu, J., Xu, Z., Zeng, Q., et al. (2019). Utilization of the Genomewide Wheat 55K SNP Array for Genetic Analysis of Stripe Rust Resistance in Common Wheat Line P9936. *Phytopathology* 109(5)**,** 819-827. doi: 10.1094/PHYTO-10-18-0388-R.

Huang, S., Zhang, L., Liang, J., Ouyang, Y., Yan, Y., Ju, H., et al. (2025). Genome-based mutant RNA mapping identifies an NLR pair underlying *Yr6*-mediated stripe rust resistance in wheat. *Plant Commun***,** 101542. doi: 10.1016/j.xplc.2025.101542.

Huang, S., Zhang, Y., Ren, H., Li, X., Zhang, X., Zhang, Z., et al. (2022). Epistatic interaction effect between chromosome 1BL (*Yr29*) and a novel locus on 2AL facilitating resistance to stripe rust in Chinese wheat Changwu 357-9. *Theor Appl Genet* 135(7)**,** 2501-2513. doi: 10.1007/s00122-022-04133-9.

Jambuthenne, D.T., Riaz, A., Athiyannan, N., Alahmad, S., Ng, W.L., Ziems, L., et al. (2022). Mining the Vavilov wheat diversity panel for new sources of adult plant resistance to stripe rust. *Theor Appl Genet* 135(4)**,** 1355-1373. doi: 10.1007/s00122-022-04037-8.

Jia, M., Yang, L., Zhang, W., Rosewarne, G., Li, J., Yang, E., et al. (2020). Genome-wide association analysis of stripe rust resistance in modern Chinese wheat. *BMC Plant Biol* 20(1)**,** 491. doi: 10.1186/s12870-020-02693-w.

Jiang, C., Wang, X., Chen, W., Liu, T., Zhong, S., Huang, Q., et al. (2020). Resistance performance of wheat stripe rust resistance gene *Yr41* and its effect on yield parameters in F2 populations under field conditions. *Crop Protection* 134. doi: 10.1016/j.cropro.2020.105168.

Johnson, R., Stubbs, R.W., Fuchs, E., and Chamberlain, N.H. (1972). Nomenclature for physiologic races of Puccinia striiformis infecting wheat. *Transactions of the British Mycological Society* 58(3)**,** 475-480. doi: 10.1016/s0007-1536(72)80096-2.

Kanwal, M., Qureshi, N., Gessese, M., Forrest, K., Babu, P., Bariana, H., et al. (2021). An adult plant stripe rust resistance gene maps on chromosome 7A of Australian wheat cultivar Axe. *Theor Appl Genet* 134(7)**,** 2213-2220. doi: 10.1007/s00122-021-03818-x.

Klymiuk, V., Chawla, H.S., Wiebe, K., Ens, J., Fatiukha, A., Govta, L., et al. (2022). Discovery of stripe rust resistance with incomplete dominance in wild emmer wheat using bulked segregant analysis sequencing. *Commun Biol* 5(1)**,** 826. doi: 10.1038/s42003-022-03773-3.

Klymiuk, V., Wiebe, K., Chawla, H.S., Ens, J., Subramaniam, R., and Pozniak, C.J. (2025). Coordinated function of paired NLRs confers *Yr84*-mediated stripe rust resistance in wheat. *Nat Genet* 57(6)**,** 1535-1542. doi: 10.1038/s41588-025-02203-4.

Klymiuk, V., Yaniv, E., Huang, L., Raats, D., Fatiukha, A., Chen, S., et al. (2018). Cloning of the wheat *Yr15* resistance gene sheds light on the plant tandem kinase-pseudokinase family. *Nat Commun* 9(1)**,** 3735. doi: 10.1038/s41467-018-06138-9.

Krattinger, S.G., Lagudah, E.S., Spielmeyer, W., Singh, R.P., Huerta-Espino, J., McFadden, H., et al. (2009). A putative ABC transporter confers durable resistance to multiple fungal pathogens in wheat. *Science* 323(5919)**,** 1360-1363. doi: 10.1126/science.1166453.

Lan, C., Hale, I.L., Herrera-Foessel, S.A., Basnet, B.R., Randhawa, M.S., Huerta-Espino, J., et al. (2017). Characterization and Mapping of Leaf Rust and Stripe Rust Resistance Loci in Hexaploid Wheat Lines UC1110 and PI610750 under Mexican Environments. *Front Plant Sci* 8**,** 1450. doi: 10.3389/fpls.2017.01450.

Lan, C., Rosewarne, G.M., Singh, R.P., Herrera-Foessel, S.A., Huerta-Espino, J., Basnet, B.R., et al. (2014a). QTL characterization of resistance to leaf rust and stripe rust in the spring wheat line Francolin#1. *Molecular Breeding* 34(3)**,** 789-803. doi: 10.1007/s11032-014-0075-6.

Lan, C.X., Singh, R.P., Huerta-Espino, J., Calvo-Salazar, V., and Herrera-Foessel, S.A. (2014b). Genetic Analysis of Resistance to Leaf Rust and Stripe Rust in Wheat Cultivar Francolin#1. *Plant Dis* 98(9)**,** 1227-1234. doi: 10.1094/PDIS-07-13-0707-RE.

Lewellen, R.T., Sharp, E.L., and Hehn, E.R. (1967). Major and Minor Genes in Wheat for Resistance to *Puccinia Striiformis* and Their Responses to Temperature Changes. *Canadian Journal of Botany* 45(11)**,** 2155-2172. doi: 10.1139/b67-234.

Li, G., Li, J., Zhang, Y., Ma, Q., Yang, E., Zhang, P., et al. (2022). Molecular and cytogenetic dissection of stripe rust resistance gene *Yr83* from rye 6R and generation of resistant germplasm in wheat breeding. *Front Plant Sci* 13**,** 1035784. doi: 10.3389/fpls.2022.1035784.

Li, G.Q., Li, Z.F., Yang, W.Y., Zhang, Y., He, Z.H., Xu, S.C., et al. (2006). Molecular mapping of stripe rust resistance gene *YrCH42* in Chinese wheat cultivar Chuanmai 42 and its allelism with *Yr24* and *Yr26*. *Theor Appl Genet* 112(8)**,** 1434-1440. doi: 10.1007/s00122-006-0245-y.

Li, H., Wang, Q., Xu, L., Mu, J., Wu, J., Zeng, Q., et al. (2017). Rapid identification of a major effect QTL conferring adult plant resistance to stripe rust in wheat cultivar Yaco“S”. *Euphytica* 213(6). doi: 10.1007/s10681-017-1912-6.

Li, J., Dundas, I., Dong, C., Li, G., Trethowan, R., Yang, Z., et al. (2020). Identification and characterization of a new stripe rust resistance gene *Yr83* on rye chromosome 6R in wheat. *Theor Appl Genet* 133(4)**,** 1095-1107. doi: 10.1007/s00122-020-03534-y.

Li, Q., Chen, X.M., Wang, M.N., and Jing, J.X. (2011). *Yr45*, a new wheat gene for stripe rust resistance on the long arm of chromosome 3D. *Theor Appl Genet* 122(1)**,** 189-197. doi: 10.1007/s00122-010-1435-1.

Li, Y., Liu, L., Wang, M., Ruff, T., See, D.R., Hu, X., et al. (2023). Characterization and Molecular Mapping of a Gene Conferring High-Temperature Adult-Plant Resistance to Stripe Rust Originally from *Aegilops ventricosa*. *Plant Dis* 107(2)**,** 431-442. doi: 10.1094/PDIS-06-22-1419-RE.

Li, Y., Niu, Y.C., and Chen, X.M. (2009). Mapping a stripe rust resistance gene *YrC591* in wheat variety C591 with SSR and AFLP markers. *Theor Appl Genet* 118(2)**,** 339-346. doi: 10.1007/s00122-008-0903-3.

Lin, F., and Chen, X.M. (2007). Genetics and molecular mapping of genes for race-specific all-stage resistance and non-race-specific high-temperature adult-plant resistance to stripe rust in spring wheat cultivar Alpowa. *Theor Appl Genet* 114(7)**,** 1277-1287. doi: 10.1007/s00122-007-0518-0.

Lin, F., and Chen, X.M. (2008). Quantitative trait loci for non-race-specific, high-temperature adult-plant resistance to stripe rust in wheat cultivar Express. *Theor Appl Genet* 118(4)**,** 631-642. doi: 10.1007/s00122-008-0894-0.

Lin, M., Dieseth, J.A., Alsheikh, M., Yang, E., Holzapfel, J., Schurmann, F., et al. (2023). A major yellow rust resistance QTL on chromosome 6A shows increased frequency in recent Norwegian spring wheat cultivars and breeding lines. *Theor Appl Genet* 136(7)**,** 164. doi: 10.1007/s00122-023-04397-9.

Lin, X., N'Diaye, A., Walkowiak, S., Nilsen, K.T., Cory, A.T., Haile, J., et al. (2018). Genetic analysis of resistance to stripe rust in durum wheat (Triticum turgidum L. var. durum). *PLoS One* 13(9)**,** e0203283. doi: 10.1371/journal.pone.0203283.

Liu, D., Yuan, C., Singh, R.P., Randhawa, M.S., Bhavani, S., Kumar, U., et al. (2022a). Stripe rust and leaf rust resistance in CIMMYT wheat line "Mucuy" is conferred by combinations of race-specific and adult-plant resistance loci. *Front Plant Sci* 13**,** 880138. doi: 10.3389/fpls.2022.880138.

Liu, J., Chang, Z., Zhang, X., Yang, Z., Li, X., Jia, J., et al. (2013a). Putative *Thinopyrum intermedium*-derived stripe rust resistance gene *Yr50* maps on wheat chromosome arm 4BL. *Theor Appl Genet* 126(1)**,** 265-274. doi: 10.1007/s00122-012-1979-3.

Liu, L., Wang, M., Feng, J., See, D.R., and Chen, X. (2019a). Whole-Genome Mapping of Stripe Rust Resistance Quantitative Trait Loci and Race Specificity Related to Resistance Reduction in Winter Wheat Cultivar Eltan. *Phytopathology* 109(7)**,** 1226-1235. doi: 10.1094/PHYTO-10-18-0385-R.

Liu, L., Yuan, C., Wang, M., See, D.R., and Chen, X. (2020a). Mapping Quantitative Trait Loci for High-Temperature Adult-Plant Resistance to Stripe Rust in Spring Wheat PI 197734 Using a Doubled Haploid Population and Genotyping by Multiplexed Sequencing. *Front Plant Sci* 11**,** 596962. doi: 10.3389/fpls.2020.596962.

Liu, L., Yuan, C.Y., Wang, M.N., See, D.R., Zemetra, R.S., and Chen, X.M. (2019b). QTL analysis of durable stripe rust resistance in the North American winter wheat cultivar Skiles. *Theor Appl Genet* 132(6)**,** 1677-1691. doi: 10.1007/s00122-019-03307-2.

Liu, M., Zhang, C., Yuan, C., Zhang, L., Huang, L., Wu, J., et al. (2013b). Stripe Rust Resistance in Aegilops tauschii Germplasm. *Crop Science* 53(5)**,** 2014-2020. doi: 10.2135/cropsci2013.01.0008.

Liu, S., Liu, D., Zhang, C., Zhang, W., Wang, X., Mi, Z., et al. (2023). Slow stripe rusting in Chinese wheat Jimai 44 conferred by *Yr29* in combination with a major QTL on chromosome arm 6AL. *Theor Appl Genet* 136(8)**,** 175. doi: 10.1007/s00122-023-04420-z.

Liu, S., Wang, X., Zhang, Y., Jin, Y., Xia, Z., Xiang, M., et al. (2022b). Enhanced stripe rust resistance obtained by combining *Yr30* with a widely dispersed, consistent QTL on chromosome arm 4BL. *Theor Appl Genet* 135(1)**,** 351-365. doi: 10.1007/s00122-021-03970-4.

Liu, Y., Qie, Y., Li, X., Wang, M., and Chen, X. (2020b). Genome-Wide Mapping of Quantitative Trait Loci Conferring All-Stage and High-Temperature Adult-Plant Resistance to Stripe Rust in Spring Wheat Landrace PI 181410. *Int J Mol Sci* 21(2). doi: 10.3390/ijms21020478.

Long, L., Yao, F., Guan, F., Cheng, Y., Duan, L., Zhao, X., et al. (2021). A Stable Quantitative Trait Locus on Chromosome 5BL Combined with *Yr18* Conferring High-Level Adult Plant Resistance to Stripe Rust in Chinese Wheat Landrace Anyuehong. *Phytopathology* 111(9)**,** 1594-1601. doi: 10.1094/PHYTO-10-20-0465-R.

Lowe, I., Jankuloski, L., Chao, S., Chen, X., See, D., and Dubcovsky, J. (2011). Mapping and validation of QTL which confer partial resistance to broadly virulent post-2000 North American races of stripe rust in hexaploid wheat. *Theor Appl Genet* 123(1)**,** 143-157. doi: 10.1007/s00122-011-1573-0.

Lu, Y., Lan, C., Liang, S., Zhou, X., Liu, D., Zhou, G., et al. (2009). QTL mapping for adult-plant resistance to stripe rust in Italian common wheat cultivars Libellula and Strampelli. *Theor Appl Genet* 119(8)**,** 1349-1359. doi: 10.1007/s00122-009-1139-6.

Lu, Y., Wang, M., Chen, X., See, D., Chao, S., and Jing, J. (2014). Mapping of *Yr62* and a small-effect QTL for high-temperature adult-plant resistance to stripe rust in spring wheat PI 192252. *Theor Appl Genet* 127(6)**,** 1449-1459. doi: 10.1007/s00122-014-2312-0.

Luo, P., Hu, X., Zhang, H., and Ren, Z. (2009). Genes for resistance to stripe rust on chromosome 2B and their application in wheat breeding. *Progress in Natural Science* 19(1)**,** 9-15. doi: 10.1016/j.pnsc.2008.02.017.

Luo, P.G., Hu, X.Y., Ren, Z.L., Zhang, H.Y., Shu, K., and Yang, Z.J. (2008). Allelic analysis of stripe rust resistance genes on wheat chromosome 2BS. *Genome* 51(11)**,** 922-927. doi: 10.1139/G08-079.

Luo, P.G., Ren, Z.L., Zhang, H.Q., and Zhang, H.Y. (2005). Identification, Chromosome Location, and Diagnostic Markers for a New Gene (*YrCN19*) for Resistance to Wheat Stripe Rust. *Phytopathology* 95(11)**,** 1266-1270. doi: 10.1094/PHYTO-95-1266.

Lupton, F.G.H., and Macer, R.C.F. (Year). "Inheritance of resistance to yellow rust (*Puccinia glumarum* Erikss. & Henn.) in seven varieties of wheat", in: *Transactions of the British Mycological Society*), 21-45.

Ma, H., and Singh, R.P. (1996). Contribution of adult plant resistance gene *Yr18* in protecting wheat from yellow rust Plant Disease. *Europe PMC* 80(1)**,** 66-69.

Ma, J., Wingen, L.U., Orford, S., Fenwick, P., Wang, J., and Griffiths, S. (2015). Using the UK reference population Avalon x Cadenza as a platform to compare breeding strategies in elite Western European bread wheat. *Mol Breed* 35(2)**,** 70. doi: 10.1007/s11032-015-0268-7.

Maccaferri, M., Zhang, J., Bulli, P., Abate, Z., Chao, S., Cantu, D., et al. (2015). A genome-wide association study of resistance to stripe rust (*Puccinia striiformis* f. sp. *tritici*) in a worldwide collection of hexaploid spring wheat (*Triticum aestivum* L.). *G3 (Bethesda)* 5(3)**,** 449-465. doi: 10.1534/g3.114.014563.

Macer, R.C.F. (Year). "The Formal and Monosomic Genetic Analysis of Stripe Rust (*Puccinia striiformis*) Resistance in Wheat", in: *Second International Wheat Genetics Symposium*: Hereditas), 127-142.

Macer, R.C.F. (1972). The resistance of cereals to yellow rust and its exploitation by plant breeding. *Proceedings of the Royal Society of London. Series B. Biological Sciences* 181(1064)**,** 281-301. doi: 10.1098/rspb.1972.0051.

Mackenzie, A., Norman, M., Gessese, M., Chen, C., Sorensen, C., Hovmoller, M., et al. (2023). Wheat stripe rust resistance locus *Yr63* is a hot spot for evolution of defence genes - a pangenome discovery. *BMC Plant Biol* 23(1)**,** 590. doi: 10.1186/s12870-023-04576-2.

Mallard, S., Gaudet, D., Aldeia, A., Abelard, C., Besnard, A.L., Sourdille, P., et al. (2005). Genetic analysis of durable resistance to yellow rust in bread wheat. *Theor Appl Genet* 110(8)**,** 1401-1409. doi: 10.1007/s00122-005-1954-3.

Marais, G.F., Pretorius, Z.A., Wellings, C.R., McCallum, B., and Marais, A.S. (2005). Leaf rust and stripe rust resistance genes transferred to common wheat from *Triticum dicoccoides*. *Euphytica* 143(1-2)**,** 115-123. doi: 10.1007/s10681-005-2911-6.

Marchal, C., Zhang, J., Zhang, P., Fenwick, P., Steuernagel, B., Adamski, N.M., et al. (2018). BED-domain-containing immune receptors confer diverse resistance spectra to yellow rust. *Nat Plants* 4(9)**,** 662-668. doi: 10.1038/s41477-018-0236-4.

McDonald, D.B., McIntosh, R.A., Wellings, C.R., Singh, R.P., and Nelson, J.C. (2004). Cytogenetical studies in wheat XIX. Location and linkage studies on gene *Yr27* for resistance to stripe (yellow) rust. *Euphytica* 136(3)**,** 239-248. doi: 10.1023/b:Euph.0000032709.59324.45.

McIntosh, R., Mu, J., Han, D., and Kang, Z. (2018). Wheat stripe rust resistance gene *Yr24*/*Yr26*: A retrospective review. *The Crop Journal* 6(4)**,** 321-329. doi: 10.1016/j.cj.2018.02.001.

McIntosh, R.A. 2024. Catalogue of Gene Symbols for Wheat. Available: <https://wheat.pw.usda.gov/GG3/sites/default/files/data_downloads/%20Catalogue%20of%20Gene%20Symbols%20for%20Wheat%20-%202017-2024.pdf>.

McIntosh, R.A., and Lagudah, E.S. (2008). Cytogenetical studies in wheat. XVIII. Gene *Yr24* for resistance to stripe rust. *Plant Breeding* 119(1)**,** 81-93. doi: 10.1046/j.1439-0523.2000.00449.x.

McIntosh, R.A., Silk, J., and The, T.T. (1996). Cytogenetic studies in wheat XVII. Monosomic analysis and linkage relationships of gene *Yr15* for resistance to stripe rust. *Euphytica* 89(3)**,** 395-399. doi: 10.1007/bf00022298.

McIntosh, R.A., Wellings, C.R., and Park, R.F. (1995). *Wheat Rusts.* Csiro Publishing.

Metzger, R.J., and Silbaugh, B.A. (1970). Inheritance of Resistance to Stripe Rust and its Association with Brown Glume Color in Tritieum aestivum L., ‘P.I. 178383’1. *Crop Science* 10(5)**,** 567-568. doi: 10.2135/cropsci1970.0011183X001000050035x.

Moore, J.W., Herrera-Foessel, S., Lan, C., Schnippenkoetter, W., Ayliffe, M., Huerta-Espino, J., et al. (2015). A recently evolved hexose transporter variant confers resistance to multiple pathogens in wheat. *Nat Genet* 47(12)**,** 1494-1498. doi: 10.1038/ng.3439.

Mu, J., Huang, S., Liu, S., Zeng, Q., Dai, M., Wang, Q., et al. (2019). Genetic architecture of wheat stripe rust resistance revealed by combining QTL mapping using SNP-based genetic maps and bulked segregant analysis. *Theor Appl Genet* 132(2)**,** 443-455. doi: 10.1007/s00122-018-3231-2.

Naruoka, Y., Ando, K., Bulli, P., Muleta, K.T., Rynearson, S., and Pumphrey, M.O. (2016). Identification and Validation of SNP Markers Linked to the Stripe Rust Resistance Gene *Yr5* in Wheat. *Crop Science* 56(6)**,** 3055-3065. doi: 10.2135/cropsci2016.03.0189.

Ni, F., Zheng, Y., Liu, X., Yu, Y., Zhang, G., Epstein, L., et al. (2023). Sequencing trait-associated mutations to clone wheat rust-resistance gene *YrNAM*. *Nat Commun* 14(1)**,** 4353. doi: 10.1038/s41467-023-39993-2.

Nsabiyera, V., Bariana, H.S., Qureshi, N., Wong, D., Hayden, M.J., and Bansal, U.K. (2018). Characterisation and mapping of adult plant stripe rust resistance in wheat accession Aus27284. *Theor Appl Genet* 131(7)**,** 1459-1467. doi: 10.1007/s00122-018-3090-x.

Paillard, S., Trotoux-Verplancke, G., Perretant, M.R., Mohamadi, F., Leconte, M., Coedel, S., et al. (2012). Durable resistance to stripe rust is due to three specific resistance genes in French bread wheat cultivar Apache. *Theor Appl Genet* 125(5)**,** 955-965. doi: 10.1007/s00122-012-1885-8.

Pakeerathan, K., Bariana, H., Qureshi, N., Wong, D., Hayden, M., and Bansal, U. (2019). Identification of a new source of stripe rust resistance *Yr82* in wheat. *Theor Appl Genet* 132(11)**,** 3169-3176. doi: 10.1007/s00122-019-03416-y.

Pang, Y., Liu, C., Lin, M., Ni, F., Li, W., Cai, J., et al. (2022). Mapping QTL for Adult-Plant Resistance to Stripe Rust in a Chinese Wheat Landrace. *Int J Mol Sci* 23(17). doi: 10.3390/ijms23179662.

Powell, N.M., Lewis, C.M., Berry, S.T., Maccormack, R., and Boyd, L.A. (2013). Stripe rust resistance genes in the UK winter wheat cultivar Claire. *Theor Appl Genet* 126(6)**,** 1599-1612. doi: 10.1007/s00122-013-2077-x.

Pretorius, Z.A., Lan, C.X., Prins, R., Knight, V., McLaren, N.W., Singh, R.P., et al. (2016). Application of remote sensing to identify adult plant resistance loci to stripe rust in two bread wheat mapping populations. *Precision Agriculture* 18(4)**,** 411-428. doi: 10.1007/s11119-016-9461-x.

Prins, R., Pretorius, Z.A., Bender, C.M., and Lehmensiek, A. (2010). QTL mapping of stripe, leaf and stem rust resistance genes in a Kariega × Avocet S doubled haploid wheat population. *Molecular Breeding* 27(2)**,** 259-270. doi: 10.1007/s11032-010-9428-y.

Qureshi, N., Bariana, H., Forrest, K., Hayden, M., Keller, B., Wicker, T., et al. (2017). Fine mapping of the chromosome 5B region carrying closely linked rust resistance genes *Yr47* and *Lr52* in wheat. *Theor Appl Genet* 130(3)**,** 495-504. doi: 10.1007/s00122-016-2829-5.

Qureshi, N., Bariana, H.S., Zhang, P., McIntosh, R., Bansal, U.K., Wong, D., et al. (2018). Genetic Relationship of Stripe Rust Resistance Genes *Yr34* and *Yr48* in Wheat and Identification of Linked KASP Markers. *Plant Dis* 102(2)**,** 413-420. doi: 10.1094/PDIS-08-17-1144-RE.

Ramburan, V.P., Pretorius, Z.A., Louw, J.H., Boyd, L.A., Smith, P.H., Boshoff, W.H., et al. (2004). A genetic analysis of adult plant resistance to stripe rust in the wheat cultivar Kariega. *Theor Appl Genet* 108(7)**,** 1426-1433. doi: 10.1007/s00122-003-1567-7.

Randhawa, M., Bansal, U., Valarik, M., Klocova, B., Dolezel, J., and Bariana, H. (2014). Molecular mapping of stripe rust resistance gene *Yr51* in chromosome 4AL of wheat. *Theor Appl Genet* 127(2)**,** 317-324. doi: 10.1007/s00122-013-2220-8.

Randhawa, M.S., Bariana, H.S., Mago, R., and Bansal, U.K. (2015). Mapping of a new stripe rust resistance locus *Yr57* on chromosome 3BS of wheat. *Molecular Breeding* 35(2). doi: 10.1007/s11032-015-0270-0.

Ren, R.S., Wang, M.N., Chen, X.M., and Zhang, Z.J. (2012a). Characterization and molecular mapping of *Yr52* for high-temperature adult-plant resistance to stripe rust in spring wheat germplasm PI 183527. *Theor Appl Genet* 125(5)**,** 847-857. doi: 10.1007/s00122-012-1877-8.

Ren, Y., He, Z., Li, J., Lillemo, M., Wu, L., Bai, B., et al. (2012b). QTL mapping of adult-plant resistance to stripe rust in a population derived from common wheat cultivars Naxos and Shanghai 3/Catbird. *Theor Appl Genet* 125(6)**,** 1211-1221. doi: 10.1007/s00122-012-1907-6.

Ren, Y., Li, Z., He, Z., Wu, L., Bai, B., Lan, C., et al. (2012c). QTL mapping of adult-plant resistances to stripe rust and leaf rust in Chinese wheat cultivar Bainong 64. *Theor Appl Genet* 125(6)**,** 1253-1262. doi: 10.1007/s00122-012-1910-y.

Ren, Y., Liu, L.S., He, Z.H., Wu, L., Bai, B., Xia, X.C., et al. (2015). QTL mapping of adult‐plant resistance to stripe rust in a ‘Lumai 21 × Jingshuang 16’ wheat population. *Plant Breeding* 134(5)**,** 501-507. doi: 10.1111/pbr.12290.

Roder, M.S., Korzun, V., Wendehake, K., Plaschke, J., Tixier, M.H., Leroy, P., et al. (1998). A microsatellite map of wheat. *Genetics* 149(4)**,** 2007-2023. doi: 10.1093/genetics/149.4.2007.

Rosa, S.B., Zanella, C.M., Hiebert, C.W., Brule-Babel, A.L., Randhawa, H.S., Shorter, S., et al. (2019). Genetic Characterization of Leaf and Stripe Rust Resistance in the Brazilian Wheat Cultivar Toropi. *Phytopathology* 109(10)**,** 1760-1768. doi: 10.1094/PHYTO-05-19-0159-R.

Rosewarne, G.M., Herrera-Foessel, S.A., Singh, R.P., Huerta-Espino, J., Lan, C.X., and He, Z.H. (2013). Quantitative trait loci of stripe rust resistance in wheat. *Theor Appl Genet* 126(10)**,** 2427-2449. doi: 10.1007/s00122-013-2159-9.

Rosewarne, G.M., Singh, R.P., Huerta-Espino, J., Herrera-Foessel, S.A., Forrest, K.L., Hayden, M.J., et al. (2012). Analysis of leaf and stripe rust severities reveals pathotype changes and multiple minor QTLs associated with resistance in an Avocet x Pastor wheat population. *Theor Appl Genet* 124(7)**,** 1283-1294. doi: 10.1007/s00122-012-1786-x.

Rosewarne, G.M., Singh, R.P., Huerta-Espino, J., and Rebetzke, G.J. (2008). Quantitative trait loci for slow-rusting resistance in wheat to leaf rust and stripe rust identified with multi-environment analysis. *Theor Appl Genet* 116(7)**,** 1027-1034. doi: 10.1007/s00122-008-0736-0.

Rosewarne, G.M., Singh, R.P., Huerta-Espino, J., William, H.M., Bouchet, S., Cloutier, S., et al. (2006). Leaf tip necrosis, molecular markers and beta1-proteasome subunits associated with the slow rusting resistance genes *Lr46/Yr29*. *Theor Appl Genet* 112(3)**,** 500-508. doi: 10.1007/s00122-005-0153-6.

Santra, D.K., Chen, X.M., Santra, M., Campbell, K.G., and Kidwell, K.K. (2008). Identification and mapping QTL for high-temperature adult-plant resistance to stripe rust in winter wheat (*Triticum aestivum* L.) cultivar 'Stephens'. *Theor Appl Genet* 117(5)**,** 793-802. doi: 10.1007/s00122-008-0820-5.

Singh, A., Knox, R.E., DePauw, R.M., Singh, A.K., Cuthbert, R.D., Campbell, H.L., et al. (2014). Stripe rust and leaf rust resistance QTL mapping, epistatic interactions, and co-localization with stem rust resistance loci in spring wheat evaluated over three continents. *Theor Appl Genet* 127(11)**,** 2465-2477. doi: 10.1007/s00122-014-2390-z.

Singh, A., Knox, R.E., DePauw, R.M., Singh, A.K., Cuthbert, R.D., Campbell, H.L., et al. (2013). Identification and mapping in spring wheat of genetic factors controlling stem rust resistance and the study of their epistatic interactions across multiple environments. *Theor Appl Genet* 126(8)**,** 1951-1964. doi: 10.1007/s00122-013-2109-6.

Singh, R.P. (1992). Genetic Association of Leaf Rust Resistance Gene *Lr34* with Adult Plant Resistance to Stripe Rust in Bread Wheat. *Phytopathology* 82(8). doi: 10.1094/Phyto-82-835.

Singh, R.P., Mujeeb-Kazi, A., and Huerta-Espino, J. (1998). *Lr46*: a gene conferring slow-rusting resistance to leaf rust in wheat. *Phytopathology* 88(9)**,** 890-894. doi: 10.1094/PHYTO.1998.88.9.890.

Singh, R.P., Nelson, J.C., and Sorrells, M.E. (2000). Mapping *Yr28* and Other Genes for Resistance to Stripe Rust in Wheat. *Crop Science* 40(4)**,** 1148-1155. doi: 10.2135/cropsci2000.4041148x.

Singh, R.P., William, H., Huerta-Espino, J., and Crosby, M. (Year). "Identification and mapping of gene *Yr31* for resistance to stripe rust in *Triticum aestivum* cultivar Pastor", in: *10th International Wheat Genetic Symposium*, eds. Pogna NE, Romana M, Pogna EA & G. G), 411-413.

Sourdille, P., Singh, S., Cadalen, T., Brown-Guedira, G.L., Gay, G., Qi, L., et al. (2004). Microsatellite-based deletion bin system for the establishment of genetic-physical map relationships in wheat (*Triticum aestivum* L.). *Funct Integr Genomics* 4(1)**,** 12-25. doi: 10.1007/s10142-004-0106-1.

Suenaga, K., Singh, R.P., Huerta-Espino, J., and William, H.M. (2003). Microsatellite markers for genes *Lr34/Yr18* and other quantitative trait Loci for leaf rust and stripe rust resistance in bread wheat. *Phytopathology* 93(7)**,** 881-890. doi: 10.1094/PHYTO.2003.93.7.881.

Sun, Q., Wei, Y., Ni, Z., Xie, C., and Yang, T. (2008). Microsatellite marker for yellow rust resistance gene *Yr5* in wheat introgressed from spelt wheat. *Plant Breeding* 121(6)**,** 539-541. doi: 10.1046/j.1439-0523.2002.00754.x.

Sun, W., Dong, H., Liu, S., Ma, S., Zeng, Q., Li, J., et al. (2025). The wheat *Yr6* locus, allelic to *Pm5*, harbors an NLR gene pair conferring stripe rust resistance. *Plant Commun***,** 101541. doi: 10.1016/j.xplc.2025.101541.

Tabe, L., Samuel, S., Dunn, M., White, R., Mago, R., Estavillo, G., et al. (2019). Phenotypes Conferred by Wheat Multiple Pathogen Resistance Locus, *Sr2*, Include Cell Death in Response to Biotic and Abiotic Stresses. *Phytopathology* 109(10)**,** 1751-1759. doi: 10.1094/PHYTO-03-19-0099-R.

Tehseen, M.M., Tonk, F.A., Tosun, M., Randhawa, H.S., Kurtulus, E., Ozseven, I., et al. (2022). QTL Mapping of Adult Plant Resistance to Stripe Rust in a Doubled Haploid Wheat Population. *Front Genet* 13**,** 900558. doi: 10.3389/fgene.2022.900558.

Tong, J., Zhao, C., Liu, D., Jambuthenne, D.T., Sun, M., Dinglasan, E., et al. (2024). Genome-wide atlas of rust resistance loci in wheat. *Theor Appl Genet* 137(8)**,** 179. doi: 10.1007/s00122-024-04689-8.

Uauy, C., Brevis, J.C., Chen, X., Khan, I., Jackson, L., Chicaiza, O., et al. (2005). High-temperature adult-plant (HTAP) stripe rust resistance gene *Yr36* from *Triticum turgidum* ssp. *dicoccoides* is closely linked to the grain protein content locus *Gpc-B1*. *Theor Appl Genet* 112(1)**,** 97-105. doi: 10.1007/s00122-005-0109-x.

Vazquez, D.M., Peterson, J.C., Riera-Lizarazu, O., Chen, X., Heesacker, A., Ammar, K., et al. (2012). Genetic analysis of adult plant, quantitative resistance to stripe rust in wheat cultivar 'Stephens' in multi-environment trials. *Theor Appl Genet* 124(1)**,** 1-11. doi: 10.1007/s00122-011-1681-x.

Vazquez, M., Zemetra, R., Peterson, C.J., Chen, X.M., Heesacker, A., and Mundt, C.C. (2015a). Multi-location wheat stripe rust QTL analysis: genetic background and epistatic interactions. *Theor Appl Genet* 128(7)**,** 1307-1318. doi: 10.1007/s00122-015-2507-z.

Vazquez, M.D., Zemetra, R., Peterson, C.J., and Mundt, C.C. (2015b). Identification of Cephalosporium stripe resistance quantitative trait loci in two recombinant inbred line populations of winter wheat. *Theor Appl Genet* 128(2)**,** 329-341. doi: 10.1007/s00122-014-2433-5.

Wang, H., Zou, S., Li, Y., Lin, F., and Tang, D. (2020a). An ankyrin-repeat and WRKY-domain-containing immune receptor confers stripe rust resistance in wheat. *Nat Commun* 11(1)**,** 1353. doi: 10.1038/s41467-020-15139-6.

Wang, M.Y., Xu, M.R., Wang, F.T., Shah, S.J.A., Feng, J., Lin, R.M., et al. (2020b). Characterization and validation of QTLs for adult plant stripe rust resistance in Chinese wheat landrace Dabaimai. *Cereal Research Communications* 49(1)**,** 91-98. doi: 10.1007/s42976-020-00071-8.

Wang, M.Y., Xu, M.R., Wang, F.T., Shah, S.J.A., Feng, J., Lin, R.M., et al. (2021a). Characterization and validation of QTLs for adult plant stripe rust resistance in Chinese wheat landrace Dabaimai. *Cereal Research Communications* 49(1)**,** 91-98. doi: 10.1007/s42976-020-00071-8.

Wang, X., Xiang, M., Li, H., Li, X., Mu, K., Huang, S., et al. (2024). High-density mapping of durable and broad-spectrum stripe rust resistance gene *Yr30* in wheat. *Theor Appl Genet* 137(7)**,** 152. doi: 10.1007/s00122-024-04654-5.

Wang, Y., Hu, Y., Gong, F., Jin, Y., Xia, Y., He, Y., et al. (2022). Identification and Mapping of QTL for Stripe Rust Resistance in the Chinese Wheat Cultivar Shumai126. *Plant Dis* 106(4)**,** 1278-1285. doi: 10.1094/PDIS-09-21-1946-RE.

Wang, Y., Liang, F., Guan, F., Yao, F., Long, L., Zhao, X., et al. (2021b). Molecular Mapping and Analysis of an Excellent Quantitative Trait Loci Conferring Adult-Plant Resistance to Stripe Rust in Chinese Wheat Landrace Gaoxianguangtoumai. *Front Plant Sci* 12**,** 756557. doi: 10.3389/fpls.2021.756557.

Wellings, C.R., McIntosh, R.A., and Hussain, M. (1988). A New Source of Resistance to *Puccinia striiformis* f. sp. *tritici* in Spring Wheats (*Triticum aestivum*). *Plant Breeding* 100**,** 88-96. doi: doi.org/10.1111/j.1439-0523.1988.tb00222.x.

William, M., Singh, R.P., Huerta-Espino, J., Islas, S.O., and Hoisington, D. (2003). Molecular marker mapping of leaf rust resistance gene *Lr46* and its association with stripe rust resistance gene *Yr29* in wheat. *Phytopathology* 93(2)**,** 153-159. doi: 10.1094/PHYTO.2003.93.2.153.

Worland, A.J., Petrovic, S., and Law, C.N. (1988). Genetic Analysis of Chromosome 2D of Wheat. *Plant Breeding* 100(4)**,** 247-259. doi: 10.1111/j.1439-0523.1988.tb00250.x.

Worland, A.J.a.L., C. N. (1986). Genetic Analysis of Chromosome 2D of Wheat. I. The Location of Genes Affecting Height, Day-Length Insensitivity, Hybrid Dwarfism and Yellow Rust Resistance. *Plant Breeding* 96(4)**,** 331-345.

Wu, J., Ma, S., Niu, J., Sun, W., Dong, H., Zheng, S., et al. (2025). Genomics-driven discovery of superior alleles and genes for yellow rust resistance in wheat. *Nat Genet* 57(8)**,** 2017-2027. doi: 10.1038/s41588-025-02259-2.

Wu, J., Wang, Q., Kang, Z., Liu, S., Li, H., Mu, J., et al. (2017). Development and Validation of KASP-SNP Markers for QTL Underlying Resistance to Stripe Rust in Common Wheat Cultivar P10057. *Plant Dis* 101(12)**,** 2079-2087. doi: 10.1094/PDIS-04-17-0468-RE.

Wu, J., Wang, X., Chen, N., Yu, R., Yu, S., Wang, Q., et al. (2020). Association Analysis Identifies New Loci for Resistance to Chinese Yr26-Virulent Races of the Stripe Rust Pathogen in a Diverse Panel of Wheat Germplasm. *Plant Dis* 104(6)**,** 1751-1762. doi: 10.1094/PDIS-12-19-2663-RE.

Wu, L., Xia, X., Rosewarne, G.M., Zhu, H., Li, S., Zhang, Z., et al. (2015). Stripe rust resistance gene *Yr18* and its suppressor gene in Chinese wheat landraces. *Plant Breeding* 134(6)**,** 634-640. doi: 10.1111/pbr.12311.

Xiang, C., Feng, J.Y., Wang, M.N., Chen, X.M., See, D.R., Wan, A.M., et al. (2016). Molecular Mapping of Stripe Rust Resistance Gene *Yr76* in Winter Club Wheat Cultivar Tyee. *Phytopathology* 106(10)**,** 1186-1193. doi: 10.1094/PHYTO-01-16-0045-FI.

Xu, L.S., Wang, M.N., Cheng, P., Kang, Z.S., Hulbert, S.H., and Chen, X.M. (2013). Molecular mapping of *Yr53*, a new gene for stripe rust resistance in durum wheat accession PI 480148 and its transfer to common wheat. *Theor Appl Genet* 126(2)**,** 523-533. doi: 10.1007/s00122-012-1998-0.

Yan, G.P., Chen, X.M., Line, R.F., and Wellings, C.R. (2003). Resistance gene-analog polymorphism markers co-segregating with the *Yr5* gene for resistance to wheat stripe rust. *Theor Appl Genet* 106(4)**,** 636-643. doi: 10.1007/s00122-002-1109-8.

Yan, X., Zheng, H., Zhang, P., Weldu, G.T., Li, Z., and Liu, D. (2021). QTL mapping of adult plant resistance to stripe rust in the Fundulea 900 × Thatcher RIL population. *Czech Journal of Genetics and Plant Breeding* 57(1)**,** 1-8. doi: 10.17221/71/2020-cjgpb.

Yang, E.N., Rosewarne, G.M., Herrera-Foessel, S.A., Huerta-Espino, J., Tang, Z.X., Sun, C.F., et al. (2013). QTL analysis of the spring wheat "Chapio" identifies stable stripe rust resistance despite inter-continental genotype x environment interactions. *Theor Appl Genet* 126(7)**,** 1721-1732. doi: 10.1007/s00122-013-2087-8.

Yildirim, A., Jones, S.S., Murray, T.D., and Line, R.F. (2000). Evaluation of *Dasypyrum villosum* Populations for Resistance to Cereal Eyespot and Stripe Rust Pathogens. *Plant Dis* 84(1)**,** 40-44. doi: 10.1094/PDIS.2000.84.1.40.

Yuan, F.P., Zeng, Q.D., Wu, J.H., Wang, Q.L., Yang, Z.J., Liang, B.P., et al. (2018). QTL Mapping and Validation of Adult Plant Resistance to Stripe Rust in Chinese Wheat Landrace Humai 15. *Front Plant Sci* 9**,** 968. doi: 10.3389/fpls.2018.00968.

Zeng, Q., Wu, J., Huang, S., Yuan, F., Liu, S., Wang, Q., et al. (2019a). SNP-based linkage mapping for validation of adult plant stripe rust resistance QTL in common wheat cultivar Chakwal 86. *The Crop Journal* 7(2)**,** 176-186. doi: 10.1016/j.cj.2018.12.002.

Zeng, Q., Wu, J., Liu, S., Chen, X., Yuan, F., Su, P., et al. (2019b). Genome-wide Mapping for Stripe Rust Resistance Loci in Common Wheat Cultivar Qinnong 142. *Plant Dis* 103(3)**,** 439-447. doi: 10.1094/PDIS-05-18-0846-RE.

Zhang, C., Huang, L., Zhang, H., Hao, Q., Lyu, B., Wang, M., et al. (2019a). An ancestral NB-LRR with duplicated 3'UTRs confers stripe rust resistance in wheat and barley. *Nat Commun* 10(1)**,** 4023. doi: 10.1038/s41467-019-11872-9.

Zhang, P., Lan, C., Singh, R.P., Huerta-Espino, J., Li, Z., Lagudah, E., et al. (2022). Identification and Characterization of Resistance Loci to Wheat Leaf Rust and Stripe Rust in Afghan Landrace "KU3067". *Front Plant Sci* 13**,** 894528. doi: 10.3389/fpls.2022.894528.

Zhang, P., Li, X., Gebrewahid, T.W., Liu, H., Xia, X., He, Z., et al. (2019b). QTL Mapping of Adult-Plant Resistance to Leaf and Stripe Rust in Wheat Cross SW 8588/Thatcher using the Wheat 55K SNP Array. *Plant Dis* 103(12)**,** 3041-3049. doi: 10.1094/PDIS-02-19-0380-RE.

Zhang, P., Yan, X., Gebrewahid, T.W., Zhou, Y., Yang, E., Xia, X., et al. (2021). Genome-wide association mapping of leaf rust and stripe rust resistance in wheat accessions using the 90K SNP array. *Theor Appl Genet* 134(4)**,** 1233-1251. doi: 10.1007/s00122-021-03769-3.

Zhang, Q., Zhang, L., Zhu, W., and Xie, S. (1998). Utilization of *Haynaldia villosa* (L.) Shur. in resistance breeding in wheat. *Acta Phytophylacica Sinica* 25(1)**,** 41-45.

Zhou, C., Liu, D., Zhang, X., Wu, Q., Liu, S., Zeng, Q., et al. (2022a). Combined linkage and association mapping reveals two major QTL for stripe rust adult plant resistance in Shaanmai 155 and their haplotype variation in common wheat germplasm. *The Crop Journal* 10(3)**,** 783-792. doi: 10.1016/j.cj.2021.09.006.

Zhou, X., Hu, T., Li, X., Yu, M., Li, Y., Yang, S., et al. (2019). Genome-wide mapping of adult plant stripe rust resistance in wheat cultivar Toni. *Theor Appl Genet* 132(6)**,** 1693-1704. doi: 10.1007/s00122-019-03308-1.

Zhou, X., Li, X., Han, D., Yang, S., Kang, Z., and Ren, R. (2022b). Genome-Wide QTL Mapping for Stripe Rust Resistance in Winter Wheat Pindong 34 Using a 90K SNP Array. *Front Plant Sci* 13**,** 932762. doi: 10.3389/fpls.2022.932762.

Zhou, X., Zhong, X., Roter, J., Li, X., Yao, Q., Yan, J., et al. (2021). Genome-Wide Mapping of Loci for Adult-Plant Resistance to Stripe Rust in Durum Wheat Svevo Using the 90K SNP Array. *Plant Dis* 105(4)**,** 879-888. doi: 10.1094/PDIS-09-20-1933-RE.

Zhou, X.L., Han, D.J., Chen, X.M., Gou, H.L., Guo, S.J., Rong, L., et al. (2014a). Characterization and molecular mapping of stripe rust resistance gene *Yr61* in winter wheat cultivar Pindong 34. *Theor Appl Genet* 127(11)**,** 2349-2358. doi: 10.1007/s00122-014-2381-0.

Zhou, X.L., Han, D.J., Chen, X.M., Mu, J.M., Xue, W.B., Zeng, Q.D., et al. (2015). QTL mapping of adult-plant resistance to stripe rust in wheat line P9897. *Euphytica* 205(1)**,** 243-253. doi: 10.1007/s10681-015-1447-7.

Zhou, X.L., Wang, M.N., Chen, X.M., Lu, Y., Kang, Z.S., and Jing, J.X. (2014b). Identification of *Yr59* conferring high-temperature adult-plant resistance to stripe rust in wheat germplasm PI 178759. *Theor Appl Genet* 127(4)**,** 935-945. doi: 10.1007/s00122-014-2269-z.

Zhu, Z., Cao, Q., Han, D., Wu, J., Wu, L., Tong, J., et al. (2023). Molecular characterization and validation of adult-plant stripe rust resistance gene *Yr86* in Chinese wheat cultivar Zhongmai 895. *Theor Appl Genet* 136(6)**,** 142. doi: 10.1007/s00122-023-04374-2.

Zou, J., Semagn, K., Chen, H., Iqbal, M., Asif, M., N’Diaye, A., et al. (2017). Mapping of QTLs associated with resistance to common bunt, tan spot, leaf rust, and stripe rust in a spring wheat population. *Molecular Breeding* 37(12). doi: 10.1007/s11032-017-0746-1.
